# Fibrosis Protein-Protein Interactions from Google Matrix Analysis of MetaCore Network

**DOI:** 10.3390/ijms23010067

**Published:** 2021-12-22

**Authors:** Ekaterina Kotelnikova, Klaus M. Frahm, Dima L. Shepelyansky, Oksana Kunduzova

**Affiliations:** 1 Clarivate Analytics, 08025 Barcelona, Spain; Ekaterina.Kotelnikova@Clarivate.com; 2 Laboratoire de Physique Théorique, IRSAMC, Université de Toulouse, CNRS, UPS, 31062 Toulouse, France; frahm@irsamc.ups-tlse.fr; 3 National Institute of Health and Medical Research (INSERM) U1048, CEDEX 4, 31432 Toulouse, France; oxana.koundouzova@inserm.fr; 4 Institute of Metabolic and Cardiovascular Diseases, University of Toulouse, UPS, 31062 Toulouse, France

**Keywords:** fibrosis, Markov chains, Google matrix, directed networks, protein–protein interactions

## Abstract

Protein–protein interactions is a longstanding challenge in cardiac remodeling processes and heart failure. Here, we use the MetaCore network and the Google matrix algorithms for prediction of protein–protein interactions dictating cardiac fibrosis, a primary cause of end-stage heart failure. The developed algorithms allow identification of interactions between key proteins and predict new actors orchestrating fibroblast activation linked to fibrosis in mouse and human tissues. These data hold great promise for uncovering new therapeutic targets to limit myocardial fibrosis.

## 1. Introduction

Cardiovascular disease, a class of diseases that impact the cardiovascular system, is responsible for 31% of all deaths and remains the leading cause of mortality worldwide [1]. Myocardial fibrosis is central to the pathology of cardiovascular complications that leads to human failure and death [2]. Cardiac fibrosis results from uncontrolled fibroblast activity and excessive extracellular matrix deposition [2]. Although a number of factors have been implicated in orchestrating the fibrotic response, tissue fibrosis is dominated by a central mediator: transforming growth factor-β (TGF-β) [3]. Sustained TGF-β production leads to a continuous cycle of growth factor signaling and deregulated matrix turnover [3]. However, despite intensive research, the factors that orchestrate fibrosis are still poorly understood and, as a result, effective strategies for reversing fibrosis are lacking [2,4]. Considering the complex heterogeneity of fibrosis, research strategy on a system-level understanding of the disease using mathematical modeling approaches is a driving force to dissect the complex processes involved in fibrotic disorders. Recently, we have reproduced the classic hallmarks of aberrant cardiac fibroblast activation leading to fibrosis, and provided a powerful toolbox for fully characterizing cardiac fibroblast transcriptome [5]. Although the pathogenesis of fibrotic remodeling has not been well identified, accumulated evidence suggests that multiple genes/proteins and their interactions play important roles in disease scenarios [6].

Traditional research has been performed to reveal the involvement of a particular gene or protein in fibrosis physiopathology [5,7]. Although these studies generated invaluable data, they still provide a small amount of evidence that is insufficient to clarify the complex nature of interactions between multiple genes or proteins simultaneously. Consequently, it is essential to develop new, multitiered approaches for global analysis of molecular interactions defining cell functional status in pathological conditions. In this context, protein–protein interactions (PPI) represent a highly promising, although challenging, class of potential targets for therapeutic development. The PPI control key functions and physio(patho)logical states of the cells. In fibrotic tissue remodeling, PPI form signaling nodes and hubs that transmit pathophysiological cues along molecular networks to achieve an integrated biological output, thereby promoting fibrosis [6]. Thus, pathway perturbation, through disruption of PPI critical for fibrosis, offers a novel and effective strategy for curtailing the transmission of profibrotic signals. Deciphering of fibrosis-specific PPI would uncover new mechanisms of fibrotic signaling for therapeutic interrogation.

In this study, we propose a Google matrix-based approach for the prediction of PPI linked to myocardial fibrosis using MetaCore network database. The present work is based on the recent results presented in [5] which allowed determination of the protein profibrotic responses as a feedback on TGF protein stimulation, which is known to play an important role in tissue fibrosis [3]. These experiments identify proteins with most positive and most negative response in cardiac fibroblasts.

To sum up, from the experimental results reported in [5], we select 40 proteins, including the top 20 positive and top 20 negative responses. The protein profile is given in Table 1 marked by indexes $K_{u}=1,2,\ldots ,20;K_{d}=1,2,\ldots ,20$. These proteins are ordered monotonically from the strongest $K_{u}=1$ to to weakest $K_{u}=20$ positive responses; the same monotonic ordering is performed by modulus of negative response with strongest $K_{d}=1$ to weakest $K_{d}=20$ responses. An additional group of 4 TGF-β-associated proteins with indexes $K_{t}=1,2,3,4$ was integrated in the primary list of factors used in experiments [5]. These 44 proteins form the internal selected fibrosis group. For the analysis of PPI characterizing fibrosis, we added a group of 10 external proteins with indexes $K_{x}=1,2,\ldots ,10$. The choice of these 10 proteins is explained below in detail, but in short, these external proteins are those which affect, according to our network analysis, the internal proteins in the strongest manner. Thus, in total we have the PPI fibrosis network with 54 proteins (nodes). They are ordered by their global index $K_{g}=1,2,\ldots ,54$ in Table 1 (first 4 $K_{t}$, then 20 $K_{u}$, 20 $K_{d}$ and 10 $K_{x}$).

To analyze the properties of this PPI fibrosis network, we use the developed commercial MetaCore network database of Clarivate [8]. This network database has been shown to be useful for analysis of various specific biological problems (see, e.g., [9,10]). At present, the MetaCore network has *N* = 40,079 nodes with $N_{\ell }$ = 292,191 links (without self-connections) with on average $n_{\ell }=N_{\ell }/N\approx 7.3$ links per node [11]. The nodes are given mainly by proteins but there are also certain molecules and molecular clusters catalyzing the interactions with proteins. This MetaCore PPI network is directed and nonweighted. In addition, its network links mark the bifunctional nature of interactions leading to the activation or the inhibition of one protein by another one. For some nodes, link action is neutral or unknown. Thus, overall, the MetaCore network is a network with activation or inhibition directed links showing that a protein A acts on protein B. We note that this network is based on a detailed analysis of world literature describing experimental results of how one protein acts on another one. The construction of this network has been performed during several years and is now continued at Clarivate [8]. Scientific biological results obtained with this MetaCore network can, for example, be found at [9,10]. This MetaCore network represents a commercial product actively used by the world’s leading pharmaceutic companies [8].

We note that at present, new types of computational methods are actively being developed, e.g., using DeepMind methods [12], with new possibilities of predicting new structures and interactions between proteins. Such methods appear to be very promising. Indeed, they can add new interaction links between proteins in the MetaCore network. However, the creation of such a global PPI network as MetaCore with almost all proteins requires long work of gathering all available interactions between proteins and representing these interactions in a format of directed network which is very useful for scientific analysis of multiple PPI. We note that there are also other types of PPI networks developed by other companies and research groups (e.g., TRANSPATH [13], REACTOME [14]). Here, we present a universal mathematical analysis based on Google matrix methods which can be also applied to other PPI networks, such as [13,14]. However, here, we present the analysis only for the MetaCore network available to us.

For the investigation of fibrosis PPI network, we use the Google matrix algorithms developed for the analysis of the World Wide Web [15,16] and other directed networks, such as Wikipedia networks, world trade networks, and others (see review [17]). Such an approach to network characterization is based on the concept of Markov chains invented by Markov in an article published in 1906 in the proceeding of the Kazan University [18].

The important method for analysis of directed networks is the reduced Google matrix (REGOMAX) algorithm developed and described in detail in [19,20]. The REGOMAX algorithm has been applied to PPI networks of SIGNOR database as reported in [21,22]. However, the number of nodes in the SIGNOR database is approximately ten times smaller than in the MetaCore network. Thus, the SIGNOR network can only be considered as a test bed for the numerical algorithms and its conceptional base. A first description of the statistical properties of the global MetaCore network, including PageRank, CheiRank, and REGOMAX characteristics, was presented in [11]. However, this work only represents a statistical study of the MetaCore network without any applications to a concrete biological problem. In this work, we apply the REGOMAX analysis to the specific biological problem of fibrosis.

The important feature of the REGOMAX algorithm is that it constructs the Google matrix of a selected subset of nodes $N_{r}\ll N$ (here, we have $N_{r}=54$) taking into account not only direct links between these $N_{r}$ nodes but also all indirect pathways connecting them via the global MetaCore network of much larger size *N*. The efficiency of the REGOMAX approach was demonstrated for various applications concerning the Wikipedia and world trade networks [23,24,25,26], and we also expect that this method will provide useful and new insights in the context of fibrosis protein–protein interactions using the MetaCore network.

The paper is constructed as follows: Section 2 describes the datasets and Google matrix algorithms, Section 3 presents the obtained results of the reduced Google matrix and sensitivity analysis for the particular group of 54 proteins (of Table 1) we consider here, and Section 4 provides the discussion of the results and the conclusion. In Appendix A, we provide additional figures and a simple analytical estimate for the sensitivity matrix to which we refer in the main part of the work; more detailed and additional numerical data obtained from the Google matrix computations are available at [27].

## 2. Datasets and Methods

### 2.1. Network Datasets

The global MetaCore PPI network contains *N* = 40,079 nodes with $N_{\ell }$ = 292,191 links (without self connections). The number of activation/inhibition links is $N_{\ell +}/N_{\ell -}$ = 65,157/49,321 ≃ 1.3 and the number of neutral links is $N_{\ell n}=N-N_{\ell +}-N_{\ell -}=177,713$. Here, we mainly present the results without taking into account the bifunctional nature of links. However, a part of the results takes into account this bifunctionality of links using the Ising Google matrix approach described in [11,22]. The subset of selected $N_{r}=54$ fibrosis proteins (nodes) is given in Table 1; these nodes are represented by 4 TGF-β proteins/nodes ($K_{t}=1,2,3,4$), 20 “up-proteins” ($K_{u}=1,\ldots ,20$), 20 “down-proteins” ($K_{d}=1,\ldots ,20$), both obtained from experiments [5] (as described above), and 10 new “X-proteins” (or “X-nodes”; $K_{x}=1,\ldots ,10$) whose selection is explained later. The TGF-β 4 nodes correspond to different isoforms of this protein. In Table 1, we show four groups of proteins and we consider that it is useful to use a specific index for each group: TGF-β proteins with index $K_{t}=1,2,3,4$; up-proteins with a strongest positive response noted by index $K_{u}=1,\cdots ,20$ (ordered by the positive response with the strongest response for $K_{u}=1$); down-proteins with a strongest negative response noted by index $K_{d}=1,\cdots ,20$ (ordered by the modulus of negative response with the strongest response modulus for $K_{d}=1$); external proteins noted by index $K_{x}$ ordered by their local PageRank index (strongest PageRank probability of these 10 proteins is at $K_{x}=1$; see more details below). All these 54 proteins have their global index $K_{g}=1,\cdots ,54$ as is shown in Table 1.

The Google matrix approach used in this work is explained in detail in [15,16,17], and the related REGOMAX algorithm is described in [11,19,20,22]. Below, we present a short description of these methods following mainly the presentation given in [11], keeping the same notations.

### 2.2. Without Formulas: Methods, Characteristics, and Expected Network Results

Here, we present qualitative explanations without formulas of the mathematical methods and characteristics described in the next subsections. Our aim here is to give a global view of our approach for a common reader.

We use the MetaCore directed network [8] which represents an action of a protein A on protein B in a form of a directed link (edge) for *N* = 40,079 proteins forming the network nodes (proteins). Such links are obtained on the basis of careful and detailed analysis of scientific literature about thousands of experiments of various research groups that allowed collection of information about PPI and thus generated a network database with *N* = 40,079 nodes and $N_{\ell }$ = 292,191 links.

The universal mathematical methods to analyze such networks are generic and based on the concept of Markov chains [18] and Google matrix [15,16,17]. The validity of these methods has been confirmed for various directed networks from various fields of science. Therefore, since the Google matrix analysis is based on a generic mathematical foundation, we expect that this analysis will also work efficiently for PPI networks.

The Google matrix of the global MetaCore PPI network *G* is constructed with specific rules described in [15,16,17], and the mathematical aspects of this construction are given in Section 2.3. The important property of *G* is that its application (multiplication) to an initial vector *v* preserves the probability and the normalization of this vector (sum of all vector elements) remains constant (taken to be unity). As a result of multiple multiplications of *v* by *G*, any initial vector converges in the long time limit to the steady-state distribution given by the PageRank vector *P*. The components of this vector represent the probabilities of each node (protein) in this limit. The nodes with the highest probabilities are the most influential nodes of the network (all nodes are monotonically ordered by decreasing values of the PageRank components which provides the “PageRank index” *K* such K(j)=1,2,… for nodes *j* with largest values P(j)). These nodes have typically many ingoing links and it is likely that some of these ingoing links come from other nodes that also have large PageRank values.

It is also useful to consider the same network but with the inversed direction of links. For this inverse network, the corresponding PageRank is called CheiRank vector $P^{*}$ [17] with the highest probabilities $P^{*}\left(j\right)$ for nodes *j* with the CheiRank index $K^{*}\left(j\right)=1,2,\ldots$ being the most communicative nodes with typically many outgoing links.

If we are interested in a specific selected, typically rather small, group of $N_{r}$ nodes ($N_{r}\ll N$), then the reduced Google matrix (REGOMAX) algorithm (described in Section 2.4 and Equations (2)–(5)) allows us to obtain a “reduced Google matrix” $G_{R}$ which describes effective interactions between these $N_{r}$ nodes, taking into account both direct links but also all indirect links due to pathways through the complementary network of the other $N-N_{r}\gg N_{r}$ nodes. In our study, the group of 44 nodes, given in Table 1, is selected on the basis of the experimental results for fibrosis responses obtained in [5]. In addition to these 44 fibrosis internal proteins ($1\leq K_{g}\leq 44$ in Table 1), we determine a special group of 10 external proteins ($45\leq K_{g}\leq 54$ in Table 1). These external proteins are found numerically with the following procedure: outside of the 44 proteins, we take those proteins which have at least one ingoing link to the top five positive response proteins ($5\leq K_{g}\leq 9$, $1\leq K_{u}\leq 5$) and the top five negative response proteins ($25\leq K_{g}\leq 29$, $1\leq K_{d}\leq 5$). There are 122 such external proteins, so that in total we have a group of 44+122=166 proteins (44 internal and 122 external ones). With the REGOMAX algorithm we obtain the reduced Google matrix for these 166 proteins. Then, we apply small variations of the transition matrix elements from the external 122 proteins to the 5 + 5 = 10 (top response) internal proteins with the above $K_{g}$ index values. We select the 10 external proteins which have the strongest PageRank probability changes induced by such variations (this provides a quantity called “sensitivity” which is formally defined in Section 2.6; see also the detailed procedure described in Section 2.7). In this way, we obtain the group of $N_{r}=54$ proteins of Table 1 (with $1\leq K_{g}\leq 44$ being internal and $45\leq K_{g}\leq 54$ being external proteins).

For this group of 54 proteins, we again compute the reduced Google matrix $G_{R}$ and the associated sensitivity matrix from which we numerically determine which of the 10 external proteins affect in the strongest way (highest sensitivity values) the PageRank probabilities of internal proteins participating in the fibrosis process, as found in [5].

Our REGOMAX-conjecture is that these newly discovered external proteins (which mostly affect the PageRank probabilities of internal nodes) will actually produce significant effects on the fibrosis process. We point out that such a conjecture has been well confirmed in different contexts for Wikipedia networks, world trade networks, and other networks [23,24,25,26]. However, this REGOMAX-conjecture for PPI networks is still to be verified experimentally.

The possibility to take into account the bifunctional nature (activation or inhibition) of links in the MetaCore PPI network is described in Section 2.5.

Finally, we note that the validity of the REGOMAX algorithms has been confirmed for various directed networks: the world trade network from the United Nations COMTRADE and World Trade Organization databases [25,26], world influence and impact of infectious diseases and cancers from Wikipedia networks [23,24], and PPI SIGNOR networks [21,22]. Since the REGOMAX method is based on the generic and universal mathematical features of the concept of Markov chains and Google matrix, it can be applied to various fields of science involving directed networks. Here, we apply the REGOMAX analysis to the very rich and advanced MetaCore network, taking into account the protein response results reported in [5], and we predict new potential proteins which may affect significantly the fibrosis process.

Below, we present the more formal and mathematical aspects of the REGONAX analysis qualitatively outlined above.

### 2.3. Google Matrix Construction, PageRank and CheiRank

First, we construct the Google matrix *G* of the MetaCore network for the simple case where the bifunctional nature of links is neglected. Furthermore, the directed links are nonweighted. First, one defines an adjacency matrix with elements $A_{ij}$ being equal to 1 if node *j* points to node *i*, and equal to 0 otherwise. In the next step, the stochastic matrix *S* describing the node-to-node Markov transitions is obtained by normalizing each column sum of the matrix *A* elements to unity. For dangling nodes *j* corresponding to zero columns of *A*, i.e., $A_{ij}=0$ for all nodes *i*, the corresponding elements of *S* are defined by $S_{ij}=1/N$. The stochastic matrix *S* describes a Markov process on the network: a random surfer jumps from node *j* to node *i* with the probability $S_{ij}$, therefore following the directed links. The column sum normalization $\sum_{i}S_{ij}=1$ ensures the conservation of probability. The elements of the Google matrix *G* are then defined by the standard form

(1) $$G_{ij}=\alpha S_{ij}+\left(1-\alpha \right)/N$$

where α=0.85 is the usual damping factor [15,16]. The Google matrix is also column sum normalized and now the random surfer jumps on the network in accordance with the stochastic matrix *S* with a probability α and with a complementary probability $\left(1-\alpha \right)$, to an arbitrary random node of the network. The damping factor allows escape from possible isolated communities and ensures that the Markov process converges for long times rather quickly to a uniform stationary probability distribution. The latter is given by the PageRank vector *P*, which is the right eigenvector of the Google matrix *G* corresponding to the leading eigenvalue, here, λ=1. The corresponding eigenvalue equation is then GP=P. According to the Perron–Frobenius theorem, the PageRank vector *P* has positive elements and their sum is normalized to unity. The PageRank vector element P(j) gives the probability to find the random surfer on the node *j* at the stationary state of the Markov process. Thus, all nodes can be ranked by a monotonically decreasing PageRank probability. The PageRank index K(j) gives the rank of the node *j* with the highest (lowest) PageRank probability P(j) corresponding to K(j)=1 (K(j)=N). The PageRank probability P(j) is proportional, on average, to the number of ingoing links pointing to node *j*. However, it also takes into account the “importance” (i.e., PageRank probability) of the nodes having a direct link to *j*.

We note that multiple checks, described in [16,17,23] and carried out for a variety of directed networks, including PPI networks [21,22], showed that the PageRank probabilities are stable with respect to variation of α in the range (0.5,0.95). Here, we use the traditional value α=0.85 used in [15,16,21,22].

It is also useful to consider a network obtained by the inversion of all link directions. For this inverted network, the corresponding Google matrix is denoted $G^{*}$ and the corresponding PageRank vector, called the CheiRank vector $P^{*}$, is defined such as $G^{*}P^{*}=P^{*}$. A detailed statistical analysis of the CheiRank vector can be found in [28,29] (see also [17]). Similarly to the PageRank vector, the CheiRank probability $P^{*}\left(j\right)$ is proportional, on average, to the number of outgoing links going out from node *j*. The CheiRank index $K^{*}\left(j\right)$ is also defined as the rank of the node *j* according to decreasing values of the CheiRank probability $P^{*}\left(j\right)$.

### 2.4. Reduced Google Matrix (REGOMAX)

The concept of the REGOMAX algorithm was introduced in [19] and a detailed description of the first applications to groups of political leaders having articles in Wikipedia networks (different language editions) can be found in [20]. This algorithm determines effective interactions between a selected subset of $N_{r}$ nodes enclosed in a global network of size $N\gg N_{r}$. These interactions are determined taking into account direct and all indirect transitions between $N_{r}$ nodes via all the other $N_{s}=N-N_{r}$ nodes of the global network. We note that, quite often in certain network analyses, only direct links of a subset of elected $N_{r}$ nodes are taken into account, and their indirect interactions via the global network are omitted, thus clearly missing the important interactions.

On a mathematical level, the REGOMAX approach uses ideas similar to those of the Schur complement in linear algebra (see, e.g., [30]) and quantum chaotic scattering in the field of quantum chaos and mesoscopic physics (see, e.g., [31,32]). The Schur complement was introduced by Issai Schur in 1917 (see history in [30]) and found a variety of applications. In the context of Markov chains, this approach was discussed in [33]. However, there are new elements, developed in [19,20], related to a specific matrix decomposition of the Schur complement which allows one to understand its new features and to compute efficiently (numerically) the three related matrix components in the framework of the reduced Google matrix approach for very large networks (e.g., $N∼5\times 10^{6}$ as for English Wikipedia).

We write the full Google matrix *G* of the global network in the block form

(2) $$G=\left(\begin{array}{cc} G_{\mathrm{rr}} & G_{\mathrm{rs}}\\ G_{\mathrm{sr}} & G_{\mathrm{ss}} \end{array}\right)$$

where the label “r” refers to the nodes of the reduced network, i.e., the subset of $N_{r}$ nodes, and “s” to the other $N_{s}=N-N_{r}$ nodes which form the complementary network, acting as an effective “scattering network”. The reduced Google matrix $G_{R}$ acts on the subset of $N_{r}$ nodes and has the size $N_{r}\times N_{r}$. It is defined by

(3) $$G_{R}P_{r}=P_{r}\;\;.$$

Here, $P_{r}$ is a vector of size $N_{r}$, its components are the normalized PageRank probabilities of the $N_{r}$ nodes, $P_{r}\left(j\right)=P\left(j\right)/\sum_{i=1}^{N_{r}}P\left(i\right)$. The REGOMAX approach allows one to find an effective Google matrix for the subset of $N_{r}$ nodes, keeping fixed the relative ranking probabilities between these nodes. The reduced Google matrix $G_{R}$ has the form [19,20]

(4) $$G_{R}=G_{\mathrm{rr}}+G_{\mathrm{rs}}{\left(1-G_{\mathrm{ss}}\right)}^{-1}G_{\mathrm{sr}}.$$

Furthermore, it satisfies the relation of Equation (3), and it is also column sum normalized. The reduced Google matrix $G_{R}$ can be represented as the sum of three components [19,20]:

(5) $$G_{R}=G_{\mathrm{rr}}+G_{\mathrm{pr}}+G_{\mathrm{qr}}.$$

Here, the first component, $G_{\mathrm{rr}}$, corresponds to the direct transitions between the $N_{r}$ nodes; the second component, $G_{\mathrm{pr}}$, is a matrix of rank 1 with all the columns being proportional (actually approximately equal to the reduced PageRank vector $P_{r}$); the third component, $G_{\mathrm{qr}}$, describes all the “interesting indirect pathways” passing through the global network of *G* matrix. Without going into the details, we mention here that mathematically (and also numerically), $G_{\mathrm{pr}}$ is obtained from Equation (4) by extracting the contribution of the leading eigenvector of $G_{\mathrm{ss}}$ (which is very close to the PageRank of the complementary scattering network of $N_{s}$ nodes) whose eigenvalue is close to unity but it is *not exactly* unity, as $G_{\mathrm{ss}}$ is not column normalized and there is a small escape probability from the $N_{s}$ scattering nodes to the selected subset with $N_{r}$ nodes. This eigenvector therefore dominates the matrix inverse in Equation (4) and its contribution produces the rank 1 matrix $G_{\mathrm{pr}}$, and the remaining contributions of the other eigenvectors of $G_{\mathrm{ss}}$ to the matrix inverse provide the matrix $G_{\mathrm{qr}}$ which can be efficiently computed by a rapid convergent matrix series (see [19,20] for details). This point is crucial since it allows for a highly efficient numerical evaluation of all three components of $G_{R}$ also for the case where a direct numerical computation of the matrix inverse of $\left(1-G_{\mathrm{ss}}\right)$ is not possible due to very large values of *N* (note $G_{\mathrm{ss}}$ has the size $N_{s}\times N_{s}$ with $N_{s}\approx N\gg N_{r}$). While $G_{\mathrm{pr}}$, being typically numerically dominant, has a very simple rank 1 structure, the matrix $G_{\mathrm{qr}}$ contains the most nontrivial information related to indirect hidden transitions. Actually, mathematically, both components $G_{\mathrm{pr}}$ and $G_{\mathrm{qr}}$ arise from indirect pathways through the scattering nodes (represented by the matrix inverse term in Equation (4)) but $G_{\mathrm{pr}}$ can be viewed as a uniform background generated by the long time limit (i.e., the leading eigenvector of $G_{\mathrm{ss}}$) of the effective process in the complementary scattering network. The component $G_{\mathrm{qr}}$ gives the deviations from this background and in the following when we speak of “contributions from indirect pathways”, we refer essentially to the contributions of $G_{\mathrm{qr}}$. It is possible that certain matrix elements of $G_{\mathrm{qr}}$ are negative, and if this happens, this is also important information as it indicates a reduction from the uniform background for certain links (matrix elements of $G_{R}$, $G_{\mathrm{rr}}$, and $G_{\mathrm{pr}}$ are always positive due to mathematical reasons).

Furthermore, we also define the matrix $G_{\mathrm{qr}}^{\left(\mathrm{nd}\right)}$ which is obtained from the matrix $G_{\mathrm{qr}}$ by setting its diagonal elements to zero (these elements correspond to indirect self-interactions of nodes). We consider that this matrix contains the most interesting link information, direct links, and “relevant” indirect links describing the deviations from the uniform background due to $G_{\mathrm{pr}}$. The contribution of each component is characterized by their weights $W_{R}$, $W_{\mathrm{pr}}$, $W_{\mathrm{rr}}$, $W_{\mathrm{qr}}$ ($W_{\mathrm{qr}}^{\left(\mathrm{nd}\right)}$), respectively, for $G_{R}$, $G_{\mathrm{pr}}$, $G_{\mathrm{rr}}$, $G_{\mathrm{qr}}$ ($G_{\mathrm{qr}}^{\left(\mathrm{nd}\right)}$). The weight of a matrix is given by the sum of all the matrix elements divided by its size $N_{r}$ ($W_{R}=1$ due to the column sum normalization of $G_{R}$). Examples of interesting applications and studies of reduced Google matrices associated with various directed networks are described in [21,22,23,24].

### 2.5. Bifunctional Ising MetaCore Network

To take into account the bifunctional nature (activation and inhibition) of MetaCore links, we use the approach proposed in [22] with the construction of a larger network, where each node is split into two new nodes with labels (+) and (−). These two nodes can be viewed as two Ising-spin components associated with the activation and the inhibition of the corresponding protein. In the construction of the doubled “Ising” network of proteins, each element of the initial adjacency matrix is replaced by one of the following 2×2 matrices:

(6) $$\sigma_{+}=\left(\begin{array}{cc} 1 & 1\\ 0 & 0 \end{array}\right),\;\sigma_{-}=\left(\begin{array}{cc} 0 & 0\\ 1 & 1 \end{array}\right),\;\sigma_{0}=\frac{1}{2}\;\left(\begin{array}{cc} 1 & 1\\ 1 & 1 \end{array}\right)$$

where $\sigma_{+}$ applies to “activation” links, $\sigma_{-}$ to “inhibition” links, and $\sigma_{0}$ when the nature of the interaction is “unknown” or “neutral”. For the rare cases of multiple interactions between two proteins, we use the sum of the corresponding σ-matrices which increases the weight of the adjacency matrix elements. Once the "Ising" adjacency matrix is obtained, the corresponding Google matrix is constructed in the usual way, as described above. The doubled Ising MetaCore network corresponds to $N_{I}$ = 80,158 nodes and $N_{I,\ell }$ = 939,808 links given by the nonzero entries of the used σ-matrices.

Now, the PageRank vector associated with this doubled Ising network has two components $P_{+}\left(j\right)$ and $P_{-}\left(j\right)$ for every node *j* of the simple network. Due to the particular structure of the σ-matrices (Equation (6)), one can show analytically the exact identity, $P\left(j\right)=P_{+}\left(j\right)+P_{-}\left(j\right)$, where P(j) is the PageRank of the initial single PPI network [22]. The numerical verification shows that the identity $P\left(j\right)=P_{+}\left(j\right)+P_{-}\left(j\right)$ holds up to the numerical precision $∼10^{-13}$.

As in [22], we characterize each node by its PageRank “magnetization”, given by

(7) $$M\left(j\right)=\frac{P_{+}\left(j\right)-P_{-}\left(j\right)}{P_{+}\left(j\right)+P_{-}\left(j\right)}.$$

By definition, we have −1≤M(j)≤1. Nodes with positive *M* are mainly activated nodes and those with negative *M* are mainly inhibited nodes.

In this work, the results are mainly presented for the simple network without taking into account the bifunctional nature of links. However, for an illustration, we also present some results for the bifunctional network, keeping for further studies a more detailed analysis of this case.

### 2.6. Sensitivity Derivative

The reduced Google matrix $G_{R}$ of the fibrosis network describes effective interactions between $N_{r}$ nodes, taking into account all direct and indirect pathways via the global MetaCore network.

As in [11], we determine the sensitivity of PageRank probabilities with respect to a small variation of the matrix elements of $G_{R}$. The PageRank sensitivity of the node *j* with respect to a small variation of the link b→a is defined as

(8) $$D_{\left(b\to a\right)}\left(j\right)=\frac{1}{P_{r}\left(j\right)}{\left.\frac{d{P_{r}}_{\varepsilon }\left(j\right)}{d\varepsilon }\right|}_{\varepsilon =0}=\lim_{\varepsilon \to 0}\frac{1}{\varepsilon P_{r}\left(j\right)}\left[{P_{r}}_{\varepsilon }\left(j\right)-P_{r}\left(j\right)\right]\;\;.$$

Here, for fixed values of *a* and *b*, ${P_{r}}_{\varepsilon }\left(j\right)$ is the PageRank vector computed from a perturbed matrix ${G_{R}}_{\varepsilon }$ where the elements are defined by ${G_{R}}_{\varepsilon }\left(a,b\right)=G_{R}\left(a,b\right)\left(1+\varepsilon \right)/\left[1+\varepsilon G_{R}\left(a,b\right)\right]$; ${G_{R}}_{\varepsilon }\left(c,b\right)=G_{R}\left(c,b\right)/\left[1+\varepsilon G_{R}\left(a,b\right)\right]$ if c≠a and ${G_{R}}_{\varepsilon }\left(c,d\right)=G_{R}\left(c,d\right)$ if d≠b and for arbitrary *c* (including c=a). In other words, the element $G_{R}\left(a,b\right)$, corresponding to the transition b→a, is enhanced/multiplied with (1+ε) and then the column *b* is resum-normalized by multiplying it with the factor $1/[1+\varepsilon G_{R}\left(a,b\right)]$, and all other columns d≠b are not modified. We use here an efficient algorithm described in [34] to evaluate the derivative in Equation (8) exactly without usage of finite differences (see also the Appendix A for some details on this and other related points). In the following, we consider the case where j=a and we define the “sensitivity matrix” as $D_{ab}=D_{\left(b\to a\right)}\left(a\right)$. It turns out from the numerical computations that for the cases considered here, all values of $D_{ab}$ are positive: $D_{ab}>0$ which can also be analytically understood as explained in Appendix A.

### 2.7. Determination of External X-Proteins

From the experimental results of [5], we have 44 nodes of our selected subset (see the first 44 rows of Table 1). Of course, the interactions between these nodes are very important but it is also important to determine how these 44 fibrosis proteins are influenced by external nodes. To find the most important and influential external nodes, we take five top up- and five down-proteins with $K_{u}=1,\ldots ,5$ and $K_{d}=1,\ldots ,5$ from Table 1. Then, we determine all external nodes having direct ingoing 134 links to one of these 5+5 fibrosis proteins. There are 122 such proteins (some of them have several links to these 5+5 proteins providing 134 links in total). The first 44 proteins of Table 1 together with these 122 external proteins (ordered by their PageRank index) constitute an intermediary group of size 166 for which we first compute the reduced Google matrix by Equation (4) and which we note as $G_{R}^{\left(166\right)}$, and from this the associated sensitivity matrix $D_{ab}^{\left(166\right)}$ (Equation (8)) (with j=a; see also Figure A3). Then, we compute the sum of sensitivities $D_{s}^{\left(5+5\right)}\left(b\right)=\sum_{a=5}^{9}D_{ab}^{\left(166\right)}+\sum_{a=25}^{29}D_{ab}^{\left(166\right)}$ (*a*-sum over top five up- and top five down-proteins) for b=45,…166 (new external proteins). Then, we select the top 10 external proteins *b* with highest values of $D_{s}^{\left(5+5\right)}\left(b\right)$. In the following, we call this new subgroup the subgroup of X-proteins (or X-nodes). They are given in the last 10 rows of Table 1 (for $K_{g}=45,\ldots ,54$ and $K_{x}=1,\ldots ,10$). We mention that these 10 X-proteins have index values of (1,2,3,4,6,8,10,15,27) with respect to the initial list of 122 external proteins (which were already PageRank ordered). It turns out that this procedure automatically selects 10 external nodes which have approximately the strongest PageRank values. This can be understood by the fact that the matrix $D_{ab}^{\left(166\right)}$ is roughly proportional to P(b) except for a small number of cells with strong peak values (see also Figure A3 and Appendix A for a theoretical explanation). In this way, we obtain the full subset of 54 fibrosis proteins given in Table 1. The REGOMAX analysis is performed for these 54 fibrosis proteins and, unless stated otherwise, all results for $G_{R}$, $D_{ab}$, etc., refer to this group of 54 proteins.

## 3. Results

In this section, we present the results of Google matrix analysis of fibrosis protein–protein interactions.

### 3.1. Fibrosis Proteins on PageRank–CheiRank Plane

**Figure 1:**
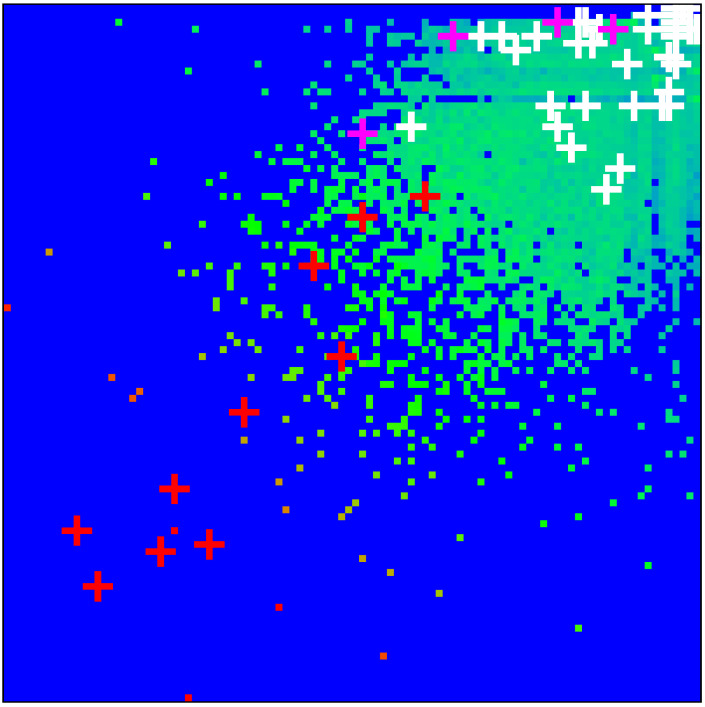
Density of nodes $W(K_{M},K_{M}^{*})$ on PageRank–CheiRank plane $\left(K_{M},K_{M}^{*}\right)$ averaged over 100×100 logarithmically equidistant grids for $0\leq \ln K_{M},\ln K_{M}^{*}\leq \ln N$ ($1\leq K_{M},K_{M}^{*}\leq N=40,079$); the density is averaged over all nodes inside each cell of the grid, the normalization condition is $\sum_{K_{M},K_{M}^{*}}W\left(K_{M},K_{M}^{*}\right)=1$. Color varies from blue at zero value to red at maximal density value. In order to increase the visibility, large density values have been reduced to (saturated at) 1/16 of the actual maximum density and typical green cells correspond to density values of $∼1/2^{8}$ of the (reduced) maximum density. The *x*-axis corresponds to $\ln K_{M}$ and the *y*-axis to $\ln K_{M}^{*}$ with $K_{M}$ ($K_{M}^{*}$) being the global PageRank (CheiRank) index for the full MetaCore network. The crosses mark the positions of the 54 proteins of Table 1 with colors: red for the X-proteins, pink for the TGF-β subgroup, and white for the up- and down-protein subgroups.

As in [11], we determine the density distribution of all proteins of the MetaCore network on the PageRank–CheiRank plane of logarithms ($\ln K,\ln K^{*})$ of indexes $\left(K,K^{*}\right)$, which is shown in Figure 1. The whole plane is divided on 100×100 logarithmically equidistant cells and the density is defined as the number of proteins in a given cell divided by a total possible nodes in a given cell (this approach is discussed in more detail, e.g., in [29]). The highest density is located at top indexes $K,K^{*}$, but in this region there is a relatively small number of proteins. The positions of fibrosis proteins of Table 1 are marked by crosses of three colors: red for 10 external X-proteins ($K_{x}=1,\ldots ,10$), pink for 4 TGF-β proteins ($K_{t}=1,2,3,4$), and white for the 40 up- and down-proteins ($K_{u},K_{d}=1,\ldots ,20$). We see that X-proteins have highest rank positions; two of the TGF-β proteins approximately follow after $K_{x}$ values of PageRank and two others have significantly lower *K*-rank positions (positions in $K^{*}$-rank are rather low); proteins $K_{u}$ and $K_{d}$ have, on average, rather low rank positions (very large $K,K^{*}$ values). Therefore the X-proteins have the highest network influence and communicativity (small $K,K^{*}$ values).

**Figure A1:**
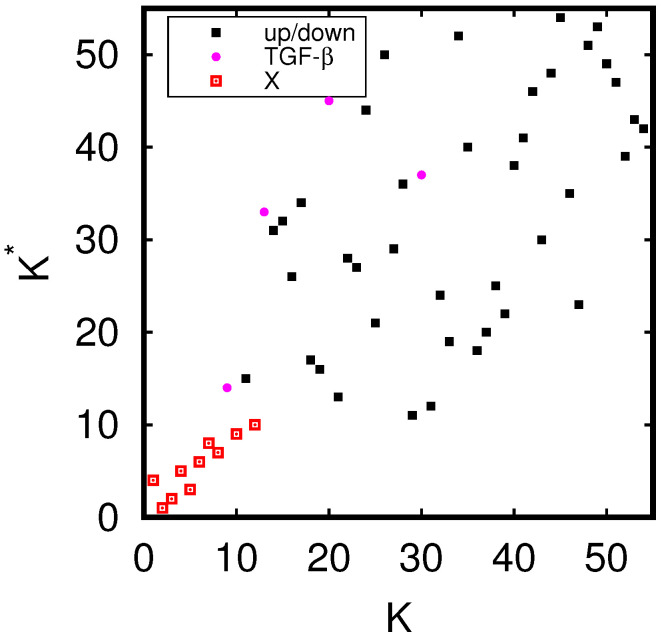
Positions of the 54 proteins of Table 1 in the local PageRank–CheiRank. Note that these positions are identical to the positions of Figure 7 and the corresponding $K,K^{*}$ values are given in the 3rd and 4th column of Table 1. Pink full circles correspond to the subgroup of TGF-β nodes, full black boxes correspond to the subgroups of up- and down-proteins and red squares correspond to the subgroup of X-proteins.

The presentation of Figure 1 uses the global MetaCore rank index values (in the following, these values are noted as $K_{M},{K^{*}}_{M}$; see also Table 1). For the selected subset of 54 fibrosis proteins, we note their local rank indexes in this group as $K,K^{*}$, which are also given in Table 1. The distribution of these 54 local rank indexes on the PageRank–CheiRank plane of size 54×54 is given in Appendix A Figure A1.

### 3.2. Reduced Google Matrix of Fibrosis

**Figure 2:**
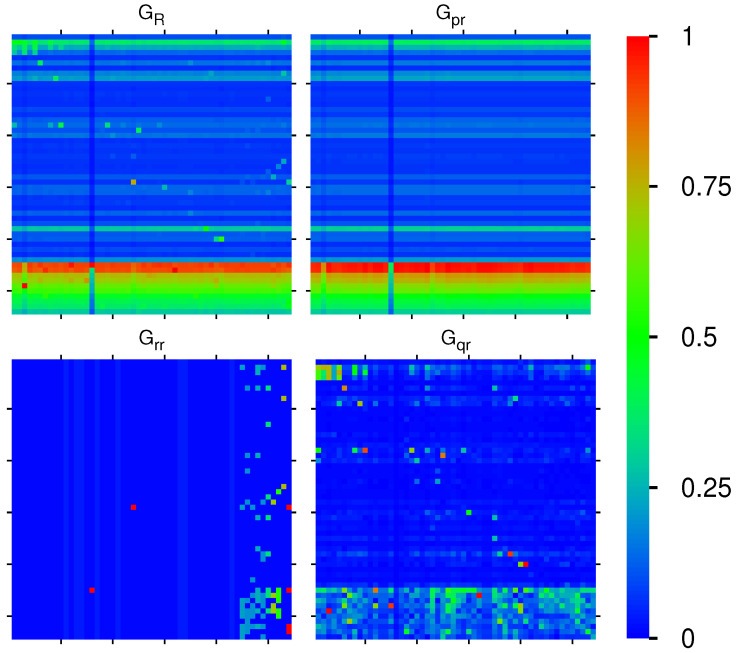
Color density plots of the matrix components $G_{R},G_{\mathrm{pr}},G_{\mathrm{rr}},G_{\mathrm{qr}}$ for the group of Table 1; the *x*-axis corresponds to the first (row) index (increasing values of $K_{g}$) from top to down) and the *y*-axis corresponds to the second (column) index of the matrix (increasing values of $K_{g}$ from left to right). The outside tics indicate multiples of 10 of $K_{g}$. The numbers in the color bar correspond to $\sqrt{\left|g\right|/g_{\max}}$, with *g* being the value of the matrix element and $g_{\max}$ being the maximum value. In order to increase the visibility for the cases of $G_{R},G_{\mathrm{rr}},G_{\mathrm{qr}}$, the maximum value has been reduced (saturated) to the value of the third largest value of *g* for each case, and the cells corresponding to the first and second largest values are reduced to the saturation value. In particular, $G_{R}\left(45,15\right)$ ($G_{R}\left(46,13\right)$) has been reduced from 0.876387 (0.297512) to $G_{R}\left(49,3\right)=0.208777$; $G_{\mathrm{rr}}\left(45,16\right)$ ($G_{\mathrm{rr}}\left(29,24\right)$) has been reduced from 0.850004 (0.121432) to $G_{\mathrm{rr}}\left(29,54\right)=0.019322$ (same third value also for the other three cells in column 54); $G_{\mathrm{qr}}\left(49,3\right)$ ($G_{\mathrm{qr}}\left(40,41\right)$) has been reduced from 0.240629 (0.062024) to $G_{\mathrm{qr}}\left(46,32\right)=0.041108$. For the matrix $G_{\mathrm{qr}}$, there are some negative values, and here, we show their absolute values (see text).

The reduced Google matrix $G_{R}$ of 54 fibrosis proteins and its 3 matrix components $G_{\mathrm{pr}},G_{\mathrm{rr}},G_{\mathrm{qr}}$ are shown in Figure 2. The weights of these matrices are: $W_{\mathrm{pr}}=0.9522$, $W_{\mathrm{rr}}=0.0228$, $W_{\mathrm{qr}}=0.0250$, ($W_{\mathrm{qr}}^{\left(\mathrm{nd}\right)}=0.0211$), and $W_{R}=1$ (due to the column sum normalization of $G_{R}$). Thus, the weight of $G_{\mathrm{pr}}$ is significantly higher compared to the two other components. This behavior is quite typical and was also observed for Wikipedia networks (see, e.g., [20,23,24]). The physical reason for this is that $G_{\mathrm{pr}}$ is obtained from the contribution of the leading eigenvector of the matrix $G_{\mathrm{ss}}$ whose eigenvalue is close to unity and dominates, numerically, the matrix inverse in Equation (4) (see also the discussion in the last section and [19,20] for details). Furthermore, $G_{\mathrm{pr}}$ has a very simple structure since it is of rank one, i.e., all columns are exact multiples of the first column. Furthermore, these columns are approximately equal to the local PageRank vector. Therefore, the component $G_{\mathrm{pr}}$ does not provide any new interesting information about possible interactions other than that it trivially reproduces the PageRank vector.

Numerically, $G_{R}$ is dominated by $G_{\mathrm{pr}}$ (with its high weight $W_{\mathrm{pr}}=0.9521$). However, the other two components give us important additional information about direct interactions between the 54 fibrosis proteins ($G_{\mathrm{rr}}$), and, even more importantly, about all indirect interactions ($G_{\mathrm{qr}}$) between these proteins via the global MetaCore network performing an effective summation over all indirect pathways (see [19,20] for details). The weights of the components of $G_{\mathrm{rr}}$ and $G_{\mathrm{qr}}$ are comparable. We also see that nearly all direct transitions visible in $G_{\mathrm{rr}}$ are from X-proteins to other proteins (all subgroups), which is not astonishing due to the selection rule that any X-node must have at least one direct link to the first five top- or first five up-proteins and also due to the fact that they have rather high PageRank but also CheiRank positions (according to Table 1, Figure 1 and Appendix A Figure A1). Since the PageRank probabilities are higher for X-proteins (see Figure 1), there are rather strong transitions to these X-proteins well visible for $G_{R}$, $G_{\mathrm{pr}}$, and, to a lesser extent, also in $G_{\mathrm{qr}}$. We note that the component $G_{\mathrm{qr}}$ has a small number of nonvanishing diagonal matrix elements which appear due to the possibility that a pathway over the global MetaCore network can return to an initial protein.

It should be noted that a few matrix elements of $G_{\mathrm{qr}}$ have negative values. Such a situation has been already found for other directed networks, e.g., Wikipedia networks studied in [20]. To be more precise for $G_{\mathrm{qr}}$ and $G_{\mathrm{rr}}+G_{\mathrm{qr}}^{\left(\mathrm{nd}\right)}$, there about 340 out of 2916 negative values (≈11%). Most of them are very small. However, there are 10 values between −0.00668 and −0.00334 for both matrices corresponding to 5–10% of the red-color saturation value used for $G_{\mathrm{qr}}$. However, in Figure 2, only the modulus of matrix elements is shown in order to have a uniform style for all components (the 10 strongest negative values of $G_{\mathrm{qr}}$ correspond to green color with color bar values of 0.3 to 0.4 and after taking the modulus). Of course, the matrix elements of $G_{R}$, $G_{\mathrm{rr}}$, and $G_{\mathrm{pr}}$ are always positive due to strict mathematical properties.

**Figure 3:**
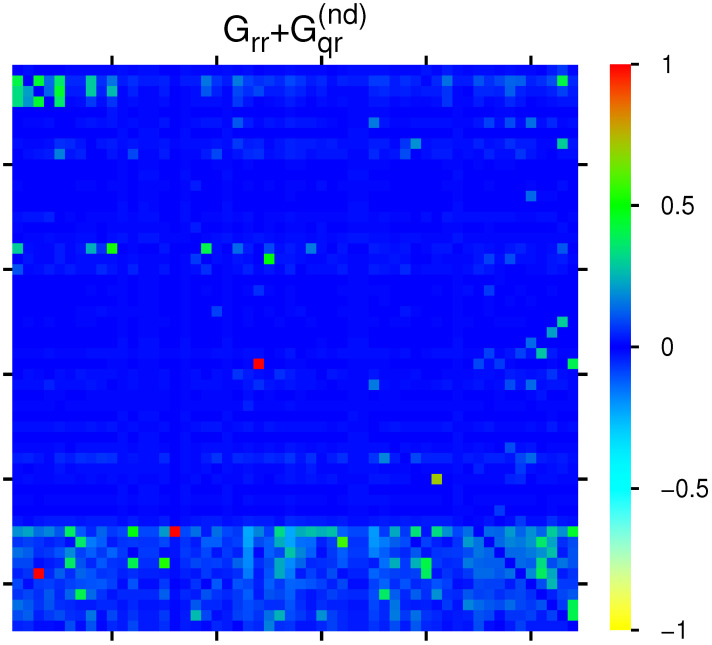
Color density plot $G_{\mathrm{rr}}+G_{\mathrm{qr}}^{\left(\mathrm{nd}\right)}$ for the group of Table 1. The matrix element at (45,16) ((49,3)) has been reduced from 0.849861 (0.240632) to the value 0.121433 at (29,24); a few matrix elements of $G_{\mathrm{rr}}+G_{\mathrm{qr}}^{\left(\mathrm{nd}\right)}$ have negative values visible as cyan color (see text). The numbers in the color bar correspond to $\mathrm{sgn}\left(g\right)\sqrt{\left|g\right|/g_{\max}}$, with *g* being the value of the matrix element and $g_{\max}$ being the maximum value.

Figure 3 shows the effective matrix of transitions for direct links and relevant indirect pathways (without self-interactions) which is obtained as the sum of the two components $G_{\mathrm{rr}}+G_{\mathrm{qr}}^{\left(\mathrm{nd}\right)}$. There are also some cells with cyan color for negative matrix elements (corresponding to −0.3 to −0.2 in units of the color bar for the strongest 10 negative values). Most links are due to the interactions from $K_{x}$ to $K_{t},K_{u},K_{d}$ proteins, but there are also some other significant transitions between the other members of the group of 54 proteins.

### 3.3. Network Diagrams of Fibrosis Interactions

In this section, we discuss two types of effective networks (of most important PPI links) obtained from the two matrices $G_{R}$ and $G_{\mathrm{rr}}+G_{\mathrm{qr}}^{\left(\mathrm{nd}\right)}$, the latter containing the “interesting” links without the uniform background generated by the component $G_{\mathrm{pr}}$ (and without self-interactions). We remind the reader that the value of a matrix element g(a,b) (with *g* being either $G_{R}$ or $G_{\mathrm{rr}}+G_{\mathrm{qr}}^{\left(\mathrm{nd}\right)}$) corresponds to the strength of the link b→a. If this value is sufficiently high, we say that *a* is a “friend” of *b* and *b* is a “follower” of *a*. This distinction allows one to construct for each matrix two types of effective networks by choosing a few number of “top nodes” and adding a certain number of the strongest friends (or followers) according to the values of |g(a,b)| and repeating this procedure for a modest number of depth levels.

**Figure 4:**
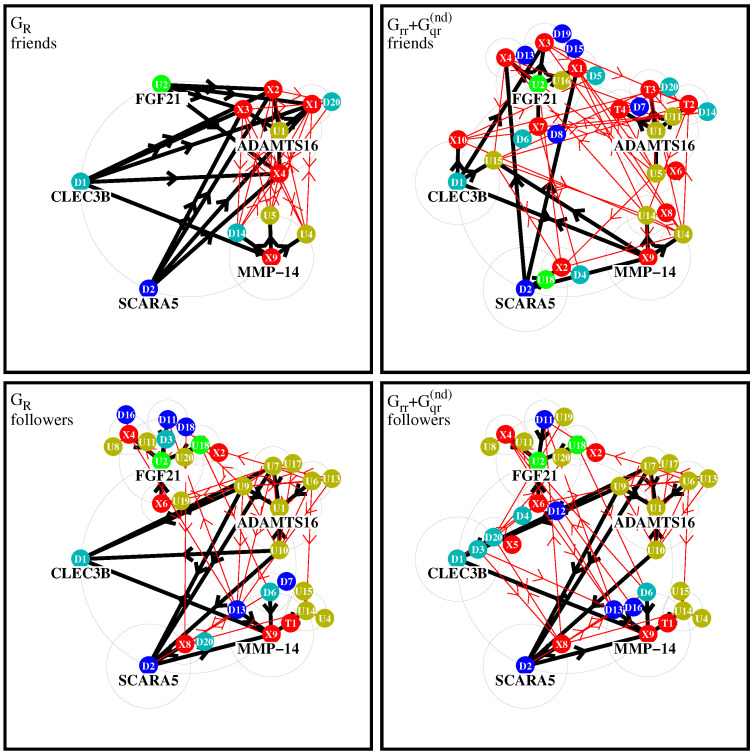
Effective friend and follower networks generated from $G_{R}$ and $G_{\mathrm{rr}}+G_{\mathrm{qr}}^{\left(\mathrm{nd}\right)}$. Starting from five top nodes, the four strongest friends/followers for each initial node are selected and links are shown by thick black arrows. For each selected new node, further four strongest friends/followers are selected and corresponding new links are shown by thin red arrows. In this procedure, the direct links between two nodes belonging both to one of the two subgroups of X-proteins or TGF-β proteins are not taken into account. The node labels Tj, Uj, Dj, Xj (with *j* being an integer value) correspond to the local subgroup index $K_{t}=j$, $K_{u}=j$, $K_{d}=j$ or $K_{x}=j$, respectively, which are given in Table 1. Color attributions: 10 external proteins $K_{x}$ and 4 TGF-β proteins are in red; protein $K_{u}=1$ and its friends are in olive green; protein $K_{u}=2$ and its friends are in green; protein $K_{d}=1$ and its friends in cyan; protein $K_{d}=2$ and its friends are in blue. Further details about precise selection rules of links, top nodes, and colors are given in the text.

In Figure 4, we show four graphical representations of such effective networks for the two cases of friend or follower networks and the two matrices $G_{R}$ and $G_{\mathrm{rr}}+G_{\mathrm{qr}}^{\left(\mathrm{nd}\right)}$ visible in Figure 2 and Figure 3. In these figures and the remainder of this subsection, we use the short notations Tj,Uj,Dj or Xj for a protein/node where j=1,2,… is the integer value of the subgroup index $K_{t},K_{u},K_{d}$ or $K_{x}$, respectively, with real protein names given in Table 1.

To construct the effective network for a matrix component *g* (with *g* being either $G_{R}$ or $G_{\mathrm{rr}}+G_{\mathrm{qr}}^{\left(\mathrm{nd}\right)}$), we first choose five initial top nodes/proteins corresponding to U1,U2 (ADAMTS16, FGF21), D1,D2 (CLEC3B, SCARA5), and X9 (MMP-14). U1,U2 (D1,D2) have the strongest positive (negative) TGF-β response observed experimentally in [5]. The node corresponding to X9 (MMP-14) produces the strongest sensitivity $D_{ab}$ (among those elements $D_{ab}$ where *a* is an up- or down protein and *b* is a TGF-β or X-protein; see next subsection for details on this). These five proteins form the set of level-0 nodes which are placed on a large circle.

We attribute the color red to the combined subgroups of 10 external X-proteins ($K_{x}=1,\ldots ,10$) and 4 TGF-β proteins ($K_{t}=1,2,3,4$). The transitions inside this red group are not taken into account since we are mainly interested in the influence of this group on the other up- and down-proteins. We attribute two colors to the up-proteins (olive green to U1, green to U2) and two colors to the down-proteins (cyan to D1, blue to D2). Inside the group of up-proteins, we attribute the color olive green to a protein Uj if Uj is a stronger follower of U1 than of U2 with respect to $g=G_{\mathrm{rr}}+G_{\mathrm{qr}}^{\left(\mathrm{nd}\right)}$, i.e., if $g\left(K_{u}=1,K_{u}=j\right)>g\left(K_{u}=2,K_{u}=j\right)$, and green otherwise. In other words, we compare the strength of the links Uj→U1 and Uj→U2 to determine if Uj has the color olive green of U1 or green of U2. In a similar way, by comparing the strength of the two links from a Dj protein to either D1 or D2, we attribute the two colors cyan and blue to down-proteins. This attribution rule, using the strongest followers with respect to $G_{\mathrm{rr}}+G_{\mathrm{qr}}^{\left(\mathrm{nd}\right)}$ of the two top nodes inside a subgroup, ensures that for all colors there is a considerable number of proteins and it is the same for all four network diagrams (both matrices and both friend/follower cases).

For each of the five level-0 proteins, noted *a*, we first search the four strongest friends (followers), noted *b*, with largest value of |g(b,a)| (or |g(a,b)|) corresponding the strongest link a→b (or b→a), where the matrix *g* is either $G_{R}$ or $G_{\mathrm{rr}}+G_{\mathrm{qr}}^{\left(\mathrm{nd}\right)}$. The new nodes *b* (if not yet present in the set of level-0 nodes) form the set of new level-1 nodes and they are placed on medium-sized circles of level 1 around the corresponding “parent” node *a* of level-0. The links between the nodes *a* and *b* are drawn as thick black arrows with direction a→b (b→a) for the friend (follower) case. If a node *b* already belongs to the set of level-0 nodes, we also draw a thick black arrow but using its already existing position on the initial large circle. If a node *b* has several parent nodes *a*, we place it only on one medium circle, preferably around a parent node of the same color if possible.

This procedure is repeated once: for each level-1 protein we determine the four strongest level-2 friends (or followers) which are placed on smaller circles of level 2 around the corresponding level-1 protein, provided that they are not yet present in the former sets of level-0 or level-1 proteins. The links corresponding to this stage are drawn as thin red arrows with the same directions as in the first stage (we also draw thin arrows for selected nodes who were already previously selected and using their former positions). As already mentioned above, links where *both* proteins (*a* and *b*) belong to the combined set of X- and TGF-β proteins are not taken into account (otherwise they would strongly dominate these diagrams). We limit ourselves to two stages of the procedure (i.e., three levels of nodes) because otherwise the diagrams would require still smaller circles and many nodes would be hidden by former nodes. We note that for the friend-$G_{R}$ diagram, a further third stage would not add any new nodes since the strongest friends of level-2 are already in the network. For the other cases, additional further stages would only add a few number of new nodes with a quite rapid saturation of the network at some limit level where no new nodes are selected.

Figure 4 shows diagrams of level-2 networks for the cases of friend (top row) and follower (bottom row) diagrams and the two matrices $g=G_{R}$ (left column) or $g=G_{\mathrm{rr}}+G_{\mathrm{qr}}^{\left(\mathrm{nd}\right)}$ (right column). Concerning the two cases of $g=G_{\mathrm{rr}}+G_{\mathrm{qr}}^{\left(\mathrm{nd}\right)}$, about 15% of the shown arrows correspond to negative values of the matrix element of *g* (link strength is determined by the modulus of the matrix element).

For the friend network of $G_{R}$, there is a dominance of links (black arrows) U1,U2,D1,D2→Xj for certain X-proteins Xj which can be understood by the fact that most Xj proteins have significantly higher PageRank probabilities than the other proteins. Furthermore, the total number of nodes in this diagram is quite small because the strongest friends of level-1 nodes (X1,X2,X3,U4,U5,D14) are mostly other level-1 nodes and there is only one new level-2 node (D20). This diagram is obviously dominated by the uniform background (of the component $G_{\mathrm{pr}}$ contributing to $G_{R}$) which tends to select mostly the “same new friends” at each level.

For the friend case of $G_{\mathrm{rr}}+G_{\mathrm{qr}}^{\left(\mathrm{nd}\right)}$, the network structure is significantly richer, since here, the global PageRank transitions (due to the uniform background of $G_{\mathrm{pr}}$) do not play a role. The group around U1 includes T2,T3,T4. Thus, we see a formation of groups of friends around U1, and especially U2, with many friends, and smaller groups of friends appear around D1,D2 and X9.

For the follower network of $G_{\mathrm{rr}}+G_{\mathrm{qr}}^{\left(\mathrm{nd}\right)}$, the largest groups of followers are again formed around U1,U2. In the group around U1, we have only other up-proteins while in the group around U2 we have up-, down-, and X-proteins. The third group around X9 is composed of several up- and down-proteins as well as one TGF-β protein (T1) on level 2. The fourth group around D1 includes D3,D20 and X5 but there are also two other followers U7, U9 which are placed on the U1-circle. The fifth group around D2 includes only X8 (on its own circle) and U7,U9,U10 from the U1-circle.

The follower network of $G_{R}$ matrix has a similar structure, since for followers the contribution of $G_{\mathrm{pr}}$ is not so significant that several links of followers of $G_{R}$ and $G_{\mathrm{rr}}+G_{\mathrm{qr}}^{\left(\mathrm{nd}\right)}$ are similar.

It should be noted that the few negative matrix elements of $G_{\mathrm{qr}}$ have a modest impact on the network diagrams of $G_{\mathrm{rr}}+G_{\mathrm{qr}}^{\left(\mathrm{nd}\right)}$ (∼15% of links and only one stage-1 link for the friend case).

These network diagrams allow us to obtain a qualitative graphical view on the most significant fibrosis PPI interactions from a friend or a follower point of view.

**Figure A2:**
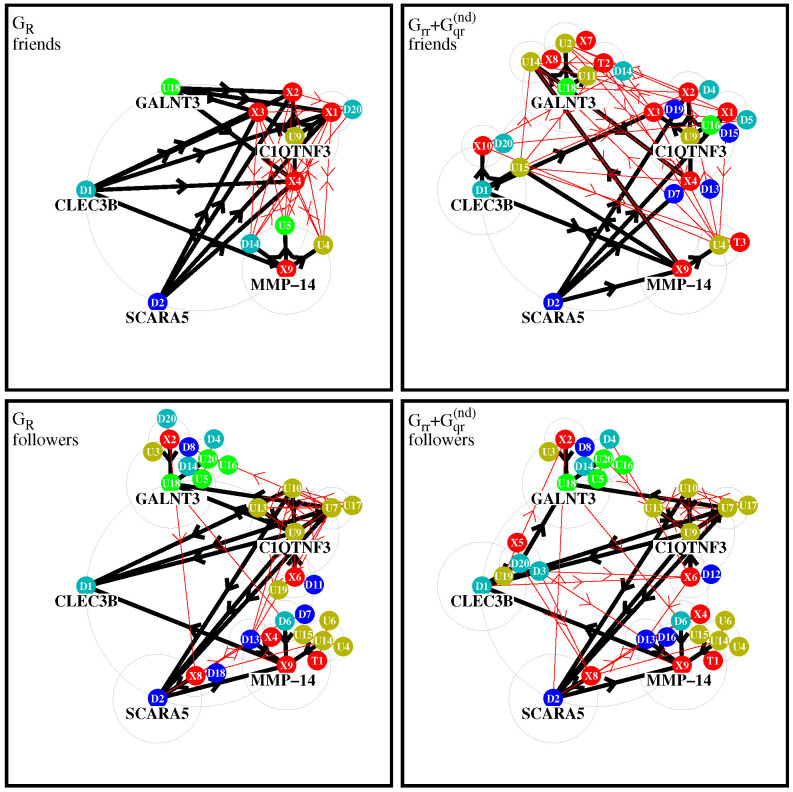
Effective network diagram for the same cases as in Figure 4 but using different 5 top nodes being the first X-node, the first two up-nodes and the first two down-nodes according to Table 2.

We note that in principle it is possible to choose another initial set of five proteins at level 0. In Appendix A Figure A2, we show the network diagrams for the modified level-0 set: D1,D2, U9,U18 and X9. Here, the four up- and down-proteins have the highest sensitivity with respect to X-proteins (see next section). Some features are quite similar to the first case: the friend diagram of $G_{R}$ has only a modest number of nodes with a domination of X-proteins, and generally, the groups associated with the two up-top nodes appear somewhat larger than the groups for the two down-top nodes.

### 3.4. Sensitivity of Fibrosis Proteins

In addition to the matrix components $G_{R},G_{\mathrm{pr}},G_{\mathrm{rr}},G_{\mathrm{qr}}$ and the network diagrams (of $G_{R}$ and $G_{\mathrm{rr}}+G_{\mathrm{qr}}^{\left(\mathrm{nd}\right)}$), it is also important to analyze the sensitivity matrix $D_{ab}$ defined previously in Equation (7). This matrix $D_{ab}$ gives the sensitivity of a protein *a* with respect to a small variation of the transition matrix element of $G_{R}$ from protein *b* to *a* on the basis of logarithmic derivative of the PageRank probability (see Section 2.5 and also Appendix A for more technical details on this).

**Figure A3:**
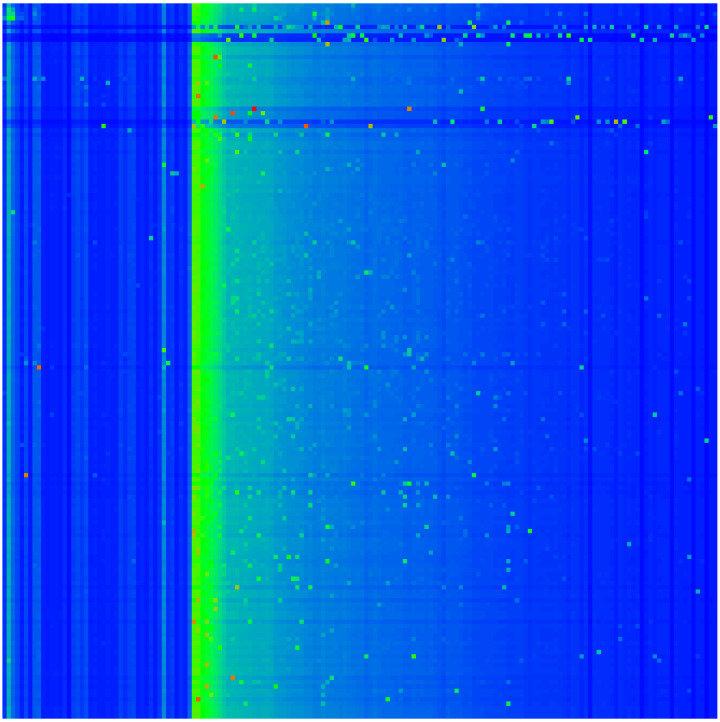
Color density plot of the sensitivity matrix $D_{ab}^{\left(166\right)}$ for the intermediary group of 166 proteins being the first 44 proteins of Table 1 (TGF-β, up- and down-subgroups) and 122 further proteins (in PageRank order) determined by having a direct link to one of the top 5 up-nodes ($K_{u}\leq 5$) or top 5 down-nodes ($K_{d}\leq 5$; see also text).

As described previously (see Section 2.6), we first compute the sensitivity matrix $D_{ab}^{\left(166\right)}$ associated with $G_{R}^{\left(166\right)}$ being the reduced Google matrix for a larger intermediary subset containing the 44 TGF-β, up- and down-proteins and further 122 external proteins having direct links (of the full MetaCore network) to the first five up- ($K_{u}=1,\ldots ,5$) and the first five down-proteins ($K_{d}=1,\ldots ,5$). This matrix is shown in Appendix A Figure A3.

Then, from the set of 122 external proteins, we select the 10 proteins *b* with the largest effective sensitivity given by the sum $D_{s}^{\left(5+5\right)}\left(b\right)=\sum_{a=5}^{9}D_{ab}^{\left(166\right)}+\sum_{a=25}^{29}D_{ab}^{\left(166\right)}$ (see Section 2.6) which form the group of 10 X-proteins. The 44 TGF-β, up- and down-proteins, together with these 10 X-proteins, form our main group of 54 proteins given Table 1 and for which we present results of the reduced Google matrix in the last subsections.

**Figure 5:**
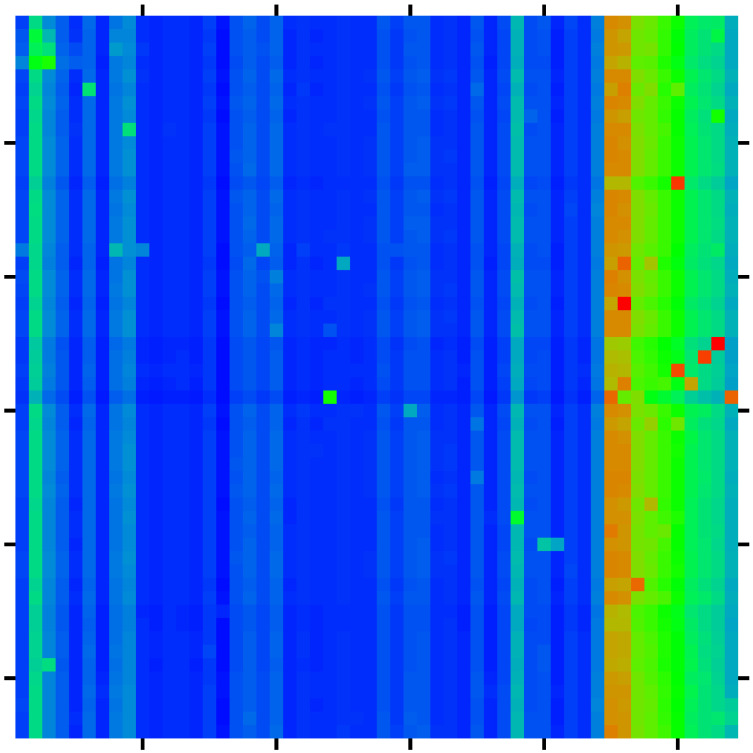
Color density plot of the sensitivity matrix $D_{ab}$ of fibrosis proteins of Table 1; the axes and colors are defined as in Figure 2 (without saturation); the strongest top 40 sensitivity values are given in Table 2.

**Figure 6:**
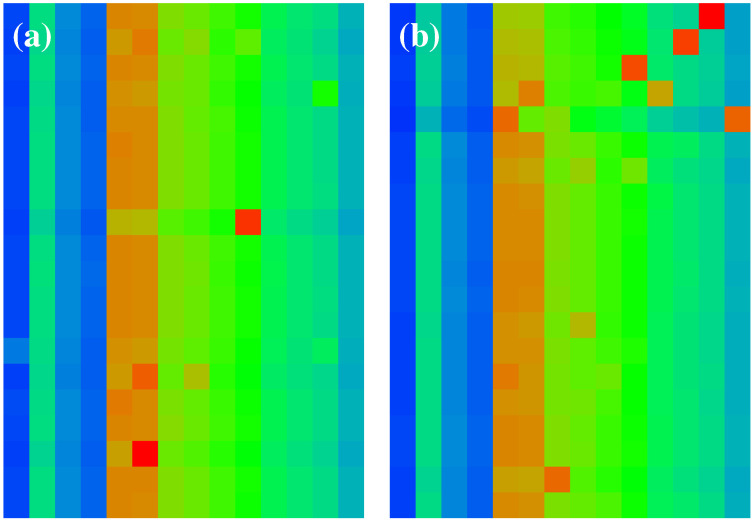
Zoomed parts of sensitivity matrix $D_{ab}$ of Figure 5. Both panels show a selected subregion of Figure 5 with the index *a* (vertical axis from top to down) belonging to the set of up-nodes (a=5,…,24 in panel (**a**)) or down-nodes (a=25,…,44 in panel (**b**)) and the index *b* (horizontal axis from left to right) corresponds to both panels to the four nodes of the TGF-β subgroup ($b=K_{t}=1,\ldots 4$ for four left columns in each panel) and the 10 nodes of the *X*-proteins (b=45,…54 or $K_{x}=1,\ldots ,10$ for 10 right columns in each panel).

The sensitivity matrix $D_{ab}$ of size 54×54 for this main group is shown in Figure 5 with zoomed parts visible in Figure 6.

The list of all 560 sensitivity matrix values $D_{ab}$ with *a* belonging to the subgroups of up- or down-proteins and *b* belonging to the subgroups of TGF-β and X-proteins is available at [27]. The strongest 40 $D_{ab}$ values of this list are shown in Table 2. Among the top three pairs, we find that the protein MMP-14 gives the top sensitivity (influence) on the protein CLEC3B ($D_{ab}=0.263109$), next is the protein p53 giving the sensitivity ($D_{ab}=0.259298$) on the protein GALNT3, and the third place is for the sensitivity of C1QTNF3 from PPAR-γ ($D_{ab}=0.225877$).

We mention that the appearance of MMP-14 ($K_{x}=9$) at the top position of Table 2 is the reason why we selected this protein as one of the five top nodes in the net diagrams discussed in the last subsection. For the net diagrams shown in Figure 4, the other four top nodes were simply chosen as the first two up- ($K_{u}=1,2$) and down-proteins ($K_{d}=1,2$). However, for the net diagrams shown in Appendix A Figure A2, the two top up- and down-nodes were also chosen by the criterion of top positions in Table 2 resulting in $K_{u}=9,18$ and $K_{d}=1,2$.

**Figure A4:**
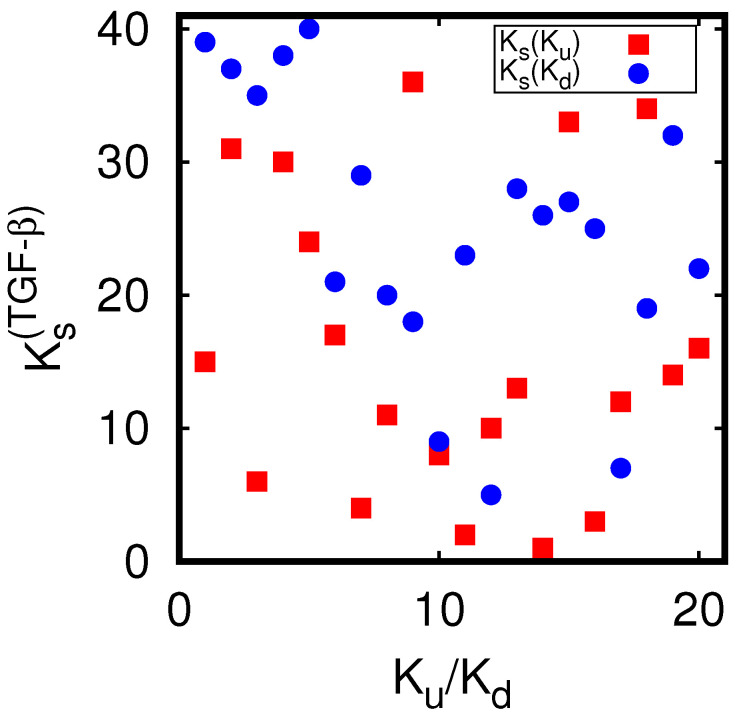
Effective ranking $K_{s}^{\left(\mathrm{TGF}-\beta \right)}$ index of the TGF-β sensitivity versus $K_{u}/K_{d}$ of up- (red boxes) and down-proteins (blue full circles). The ranking index $K_{s}^{\left(\mathrm{TGF}-\beta \right)}$ is determined by ordering the sum $D_{s}^{\left(\mathrm{TGF}-\beta \right)}\left(a\right)=\sum_{b=1}^{4}D_{ab}$ in decreasing order for a=5,…,44 (i.e., *a* belongs to one of the sets of up- or down-proteins) and where $D_{ab}$ is the sensitivity matrix for the 54 nodes of Table 1 (see also Figure 5).

We also computed the effective TGF-β sensitivity on up- or down-proteins (noted *a*) defined by the sum $D_{s}^{\left(\mathrm{TGF}-\beta \right)}\left(a\right)=\sum_{b=1}^{4}D_{ab}$. Ordering these values in decreasing order, we obtain the ranking index $K_{s}^{\left(\mathrm{TGF}-\beta \right)}=1,\ldots ,40$ whose dependence on $K_{u}$ and $K_{d}$ is visible in Appendix A Figure A4. We see that for the up-proteins we have 14 ranking values located at $K_{s}^{\left(\mathrm{TGF}-\beta \right)}\leq 20$ and for the down-proteins only 6 values at $K_{s}^{\left(\mathrm{TGF}-\beta \right)}\leq 20$ (with 3 values at $K_{s}^{\left(\mathrm{TGF}-\beta \right)}=18,19,20$). This shows that the overall influence of TGF-β proteins is somewhat stronger on the up-proteins, compared to the down-proteins.

However, we mention that the different values of $D_{s}^{\left(\mathrm{TGF}-\beta \right)}\left(a\right)$ used to determine this ranking have only modest size variations in the interval 0.0250 to 0.0465 with most values between 0.040 and 0.043. Furthermore, overall, the external X-proteins have a much higher influence (on up- and down-proteins) than the TGF-β proteins. For instance, in Table 2, the TFG-β proteins do not appear at all (in the three “*b*” columns), and in the full list of 560 entries, the first appearance of a TFG-β protein is at the ranking position $K_{s}=319$.

Both of these points can be explained by the approximate expression $D_{ab}\approx \left[1-P_{r}\left(a\right)\right]P_{r}\left(b\right)\approx P_{r}\left(b\right)$ which is derived in the appendix for a simplified model of a rank 1 $G_{R}$ matrix but which also holds approximately for arbitrary $G_{R}$ matrices due to the strong numerical weight of the rank 1 component $G_{\mathrm{pr}}$. This behavior is also confirmed, for a “uniform background”, by Figure 5 and Figure 6 for $D_{ab}$ and Appendix A Figure A3 for $D_{ab}^{\left(166\right)}$. However, there are typically some exceptional peaks at a few values of the (a,b) index pair where strong deviations from this simple expression are possible and which are due to the components of $G_{\mathrm{rr}}$ and $G_{\mathrm{qr}}$ in $G_{R}$.

Essentially, $D_{ab}∼P_{r}\left(b\right)$ does not (strongly) depend on *a*, explaining that the values of the partial sum $D_{s}^{\left(\mathrm{TGF}-\beta \right)}\left(a\right)=\sum_{b=1}^{4}D_{ab}$ show only modest size variations. Furthermore, Table 2, containing the largest $D_{ab}$ values (with *b* being either an X or a TGF-β protein and *a* being an up- or down protein), is dominated by X-proteins which have mostly larger $P_{r}\left(b\right)$ values than the TGF-β proteins.

**Figure A5:**
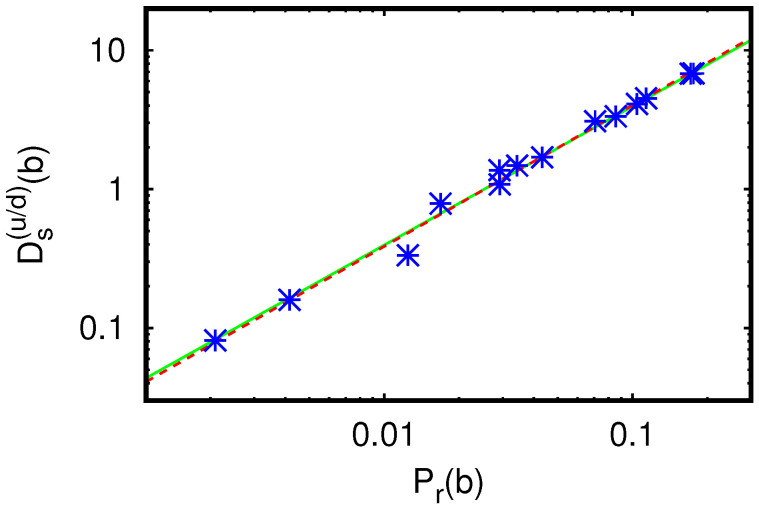
Dependence of the sum of sensitivities $D_{s}^{\left(u/d\right)}\left(b\right)$ from Table 3 on the (local) PageRank probability $P_{r}\left(b\right)$; the straight green line shows the fit dependence $D_{s}^{\left(u/d\right)}\left(b\right)=\eta P_{r}\left(b\right)$ with the obtained numerical value η=39.5±1.4; the dashed red line corresponds to the power law fit $D_{s}^{\left(u/d\right)}\left(b\right)=\tilde{\eta }{\left[P_{r}\left(b\right)\right]}^{\kappa }$ with $\tilde{\eta }=41.9\pm 4.3$ and κ=1.017±0.028.

We also determine the global influence on the whole group of fibrosis up- and down-proteins by computing the sum $D_{s}^{\left(u/d\right)}\left(b\right)=\sum_{a=5}^{44}D_{ab}$ (i.e., the *a*-sum is over up- and down-proteins) for each X or TGF-β protein *b*. The resulting values of this quantity are provided in Table 3. According to the simple expression for $D_{ab}$, we have a linear dependence of $D_{s}^{\left(u/d\right)}\left(b\right)$ on $P_{r}\left(b\right)$, and due to the *a*-su,m the effect of exceptional peaks is strongly reduced. This linear dependence is clearly visible in Table 3 and Appendix A Figure A5. A simple linear fit $D_{s}^{\left(u/d\right)}\left(b\right)=\eta P_{r}\left(b\right)$ provides the value η=39.5±1.4 for the coefficient and a more general power law fit $D_{s}^{\left(u/d\right)}\left(b\right)=\tilde{\eta }{\left[P_{r}\left(b\right)\right]}^{\kappa }$ results in a similar coefficient $\tilde{\eta }=41.9\pm 4.3$ and an exponent κ=1.017±0.028 close to unity.

However, Table 3 also shows that at the ranking positions 9 ($K_{x}=9$ for MMP-14) and 10 ($K_{t}=2$ for TGF-β 1), there is one ranking inversion between $D_{s}^{\left(u/d\right)}\left(b\right)$ and $P_{r}\left(b\right)$. The value of $D_{s}^{\left(u/d\right)}\left(K_{x}=9\right)$ is roughly 30% larger than $D_{s}^{\left(u/d\right)}\left(K_{t}=2\right)$, while the PageRank value of the former is very slightly (0.15%) smaller than the value of the latter (both PageRank values are nearly identical). In Appendix A Figure A5, both of these proteins correspond to two data points with a certain visible (vertical) difference for $D_{s}^{\left(u/d\right)}\left(b\right)$ but with no visible (horizontal) difference for $P_{r}\left(b\right)$.

We argue that the obtained high sensitivity values shown in Figure 5 and Figure 6 and Table 2 can be tested in experiments similar to those reported in [5]. The global influence $D_{s}^{\left(u/d\right)}$ from Table 3 also gives us a prediction of the globally stronger influence of the X-proteins than the TGF-β proteins. These results open new perspectives for external proteins influence on fibrosis.

### 3.5. Bifunctionality of Fibrosis Network

Here, we present in short certain results for the bifunctional MetaCore network. The doubled Ising MetaCore network has $N_{I}=$ 80,158 nodes and $N_{I,\ell }$ = 939,808 links. We compute the reduced Google matrix $G_{R}$ for the doubled number of nodes 2×54=108 (by attributing (+) and (−) labels to each node) for the fibrosis proteins of Table 1. Here, we present only some selected characteristics; all data for the Ising Google matrix are available at [27].

**Figure 7:**
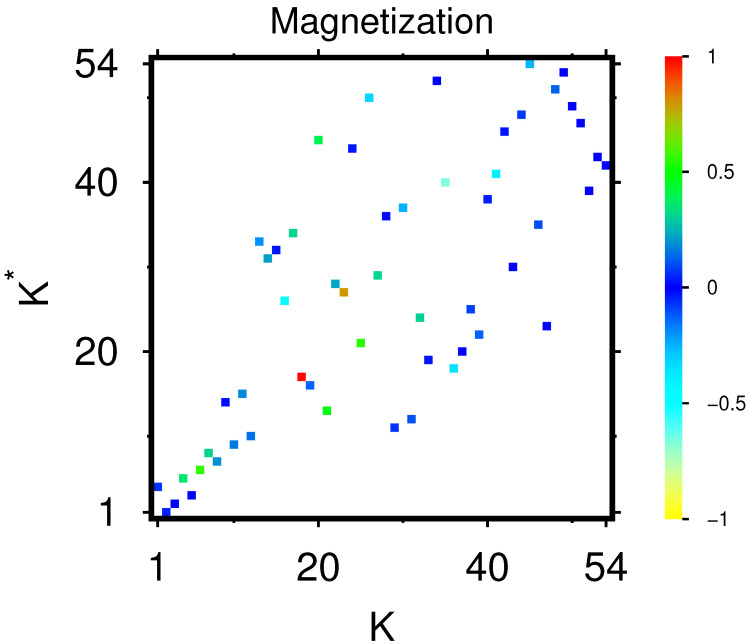
PageRank “magnetization” $M\left(j\right)=\left(P_{+}\left(j\right)-P_{-}\left(j\right)\right)/\left(P_{+}\left(j\right)+P_{-}\left(j\right)\right)$ of proteins of Table 1 shown on the PageRank–CheiRank plane $\left(K,K^{*}\right)$ of local indices; here, *j* represents a protein node in the initial single protein network and $P_{\pm }\left(j\right)$ are the PageRank components of the bifunctional Ising MetaCore network (see text). The values of the color bar correspond to M/max|M| with max|M|=0.690937 being the maximal value of |M(j)| for the shown group of proteins. Note that the positions in the PageRank–CheiRank plane are identical to the positions of Appendix A Figure A1, and the corresponding $K,K^{*}$ values are given in the third and fourth column of Table 1.

In Figure 7, we show the magnetization $M\left(j\right)=\left(P_{+}\left(j\right)-P_{-}\left(j\right)\right)/\left(P_{+}\left(j\right)+P_{-}\left(j\right)\right)$ of proteins of Table 1 with their location on the PageRank–CheiRank plane $\left(K,K^{*}\right)$. Remember that $P_{\pm }\left(j\right)$ is the PageRank value of the node *j* with label (±) and that the sum satisfies $P\left(j\right)=P_{+}\left(j\right)+P_{-}\left(j\right)$ where P(j) is the PageRank value of the node *j* of the simple network. The magnetization is positive for nodes which are more likely to be activated, or in other words, which have on average more incoming activation links (and/or coming from other nodes with larger PageRank values) than inhibition links, while negative values correspond to nodes being more likely to be inhibited by other nodes.

According to Figure 7, the majority of proteins have values of *M* being close to zero (neutral action on average coming from other nodes), but there are also some nodes with with significant positive values such as RAPGEF4 (at $K=18,K^{*}=17,K_{g}=20,K_{u}=16,M=0.690937$) corresponding to the only red box (maximum value of 1 in units of the color bar) and HMGCS2 (at $K=23,K^{*}=27,K_{g}=35,K_{d}=11,M=0.550286$) with an orange-brown box (value of 0.8 in units of the color bar). There are about a further nine proteins with various degrees of green color (*M* values between 0.2 and 0.4 corresponding to 0.3 to 0.6 in units of the color bar). The two proteins with strongest negative values of *M* are CLEC3B (at $K=35,K^{*}=40,K_{g}=25,K_{d}=1,M=-0.463912$) with a light cyan box (value of −0.7 in units of the color bar) and ACAN (at $K=16,K^{*}=26,K_{g}=8,K_{u}=4,M=-0.342585$) with a cyan box (value of −0.5 in units of the color bar). There are about five further proteins with various degrees of cyan color (*M* values between −0.28 and −0.17 corresponding to −0.4 to −0.25 in units of the color bar). We note that CELC3B is also selected in both network diagrams of Figure 4 and Appendix A Figure A2 as one of the two down-top-nodes, either because it is the first protein in the list of down-proteins or because it appears at the top position of Table 2 for the strongest sensitivity value $D_{ab}$ (with *a* being CELC3B and *b* being the X-protein MMP-14). One may also note that Appendix A Figure A1 shows the same $\left(K,K^{*}\right)$ positions as Figure 7 and allows us to identify which of the boxes belong to the subgroups of TGF-β proteins, up- or down-proteins, or X-proteins. The complete table of magnetization values used for Figure 7, including the values of $K,K^{*},K_{g}$ etc., is available in one of the data files provided in [27].

**Figure 8:**
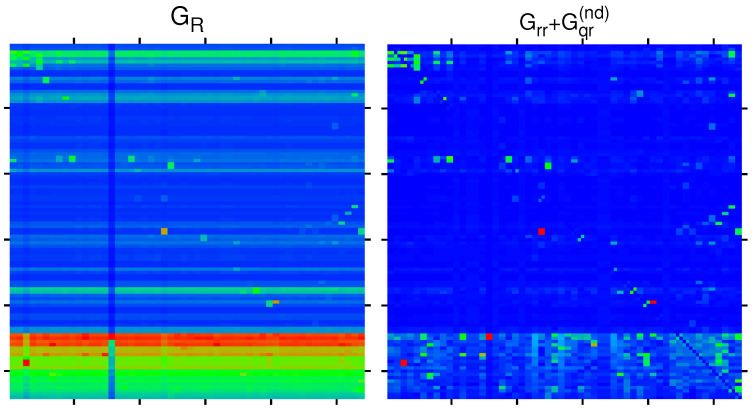
Color density plots of $G_{R}$ and $G_{\mathrm{rr}}+G_{\mathrm{qr}}^{\left(\mathrm{nd}\right)}$ for the bifunctional Ising MetaCore network and the extended group of 108 nodes by attribution of labels (+) and (−) to each node of Table 1. The matrix plot style is similar to in Figure 2, with outside tics indicating multiples of 20 of the index values. The color bar is as in Figure 2 with the same translation of colors to matrix values. The saturation value is, for both panels, the sixth largest value for each matrix, and larger values are reduced to this value. The strongest cell values are reduced from 0.437575 (0.424939) to 0.101874 (0.060717) for $G_{R}$ ($G_{\mathrm{rr}}+G_{\mathrm{qr}}^{\left(\mathrm{nd}\right)}$).

In Figure 8, we show the matrices components $G_{R}$ and $G_{\mathrm{rr}}+G_{\mathrm{qr}}^{\left(\mathrm{nd}\right)}$ for the group of selected 108 nodes corresponding to the Ising MetaCore network. Their structure is quite similar to the corresponding components for the group of 54 nodes for the simple network shown in Figure 2 and Figure 3, i.e., $G_{R}$ is dominated by the uniform background due to the component $G_{\mathrm{pr}}$ with some exceptional peak values and large values if the first (vertical) matrix index corresponds to an X-protein with large PageRank probability. For $G_{\mathrm{rr}}+G_{\mathrm{qr}}^{\left(\mathrm{nd}\right)}$, the structure is more sparse, showing the most significant direct and relevant indirect transitions. We note that for the Ising case, the matrix values are identical for the two labels of a given node in the horizontal position (except for the diagonal elements of $G_{\mathrm{rr}}+G_{\mathrm{qr}}^{\left(\mathrm{nd}\right)}$, which have been artificially set to zero), which is a mathematical property of these matrices. However, in the vertical direction, there are significant differences between the two Ising labels, especially for $G_{\mathrm{rr}}+G_{\mathrm{qr}}^{\left(\mathrm{nd}\right)}$.

Further detailed analyses of the Ising MetaCore network with applications on fibrosis interactions are kept for future studies. However, an interested reader can find additional numerical results at [27]. In particular, figures for the Ising network diagrams obtained from the Ising versions of $G_{R}$ and $G_{\mathrm{rr}}+G_{\mathrm{qr}}^{\left(\mathrm{nd}\right)}$, in the same way as in Section 3.4, are available there.

### 3.6. Summarizing Results Without Formulas

We present here a short summary of results without formulas to make them more clear for a common reader. With the REGOMAX analysis, we find the external proteins ($K_{g}=45,\ldots 54$, $K_{x}=1,\ldots 10$ in Table 1) which produce the strongest influence on the PageRank probabilities of the internal protein group ($K_{g}=5,\ldots 44$ in Table 1) characterizing the fibrosis process. Since the PageRank probabilities determine the global influence of proteins on the MetaCore PPI network, we push forward the REGOMAX-conjecture that these external proteins, found in this work, will produce a significant influence on the fibrosis process. The lists of these external proteins with their effective influence on internal proteins (sensitivity) are given in Table 2 and Table 3. We also determined the most significant interactions between the 54 fibrosis proteins; these interactions are given by their $G_{R}$ matrix elements.

We point out that such a prediction of the REGOMAX analysis has never been tested in real protein fibrosis processes. However, our previous studies of other directed networks (Wikipedia networks, world trade networks, etc. [23,24,25,26]) allowed us to compare the predictions of the REGOMAX analysis with other studies performed by other scientific methods, confirming the obtained REGOMAX results and therefore showing the efficiency of this approach. On these grounds, we expect that our predictions for fibrosis will find their experimental confirmations.

We also show that the bifunctional nature of fibrosis PPI can be also analyzed by the REGOMAX algorithm. Thus, the detailed analysis of these bifunctional effects opens unexplored perspectives left for further studies.

## 4. Conclusions

Identifying fibrosis-associated proteins is a critical issue in treating heart failure. However, deciphering fibrosis proteins experimentally is extremely time-consuming and labor-intensive. Thus, alternative methods should be developed to discover fibrosis proteins. In the current study, we explored fibroblast transcriptome profiling data [5] to develop a model for predicting cardiac fibrosis protein–protein interactions using the Google matrix analysis. Thus, we implemented the REGOMAX algorithm to the MetaCore PPI network to dissect the key proteins driving cardiac fibroblast activation leading to fibrosis.

In this work, we presented the Google matrix analysis of PPI of cardiac fibrosis. The group of 54 proteins actively participating in the fibrosis process is determined on the basis of INSERM experimental results presented in [5], which identify 44 proteins. In addition, we discover 10 external proteins with strongest sensitivity action on the fibrosis related 44-group. The sensitivity action is computed in the context of the REGOMAX approach applied to the MetaCore PPI network [8]. Our results allow us to identify the most important interactions between 54 proteins related to fibrotic cascade. The strongest integrated sensitivity actions of fibrosis proteins are summarized in Table 3, predicting the strongest influence of the myocardial fibrosis process. The strongest interactions between fibrosis proteins are also identified from the REGOMAX analysis and are summarized in Table 2.

The current research not only significantly improves the prediction performance of fibrosis proteins, but also discovers several potential fibrosis-associated proteins for future experimental investigations. It is anticipated that the current research could provide new insights into fibrosis-related disease mechanisms and diagnosis. Confirmatory testing of these predictions is planned with the experimental investigations of fibrosis to be performed at INSERM.

We argue that the developed Google matrix analysis for PPI has a generic and universal nature, being based on the strict mathematical features of Markov chains and directed networks [15,16,17,18]. Thus, this approach can be applied not only to the MetaCore network but also to other PPI network databases, such as TRANSPATH [13] and REACTOM [14]. The mathematical foundations of the Google matrix analysis have proved to be useful and efficient for different types of directed networks, including the World Wide Web [15,16], Wikipedia networks, and the world trade networks [17,20,23,25,26]. Thus, we expect that the analysis of the existing PPI network databases [8,13,14] with the Google matrix algorithms described here will find broad applications for analysis of various complex biosystems and diseases.

## Figures and Tables

**Table 1:** Table of the subset of $N_{r}=54$ selected fibrosis proteins (nodes). Here, $K_{g}$ represents the global index of this group, $K_{t,u,d,x}$ represent the index of the four subgroups of 4 TFG-β proteins, 20 up-proteins, 20 down-proteins and 10 additional X-proteins; *K* ($K^{*}$) represents the local PageRank (CheiRank) index obtained from the reduced Google matrix $G_{R}$ (${G_{R}}^{*}$) for this group of 54 proteins; $K_{M}$ ($K_{M}^{*}$) indexes represent the PageRank (CheiRank) index for the global MetaCore network of *N* = 40,079 nodes; the last column gives the associated protein names.

$K_{g}$	$K_{t,u,d,x}$	*K*	$K^{*}$	$K_{M}$	$K_{M}^{*}$	Protein
1	$K_{t}=1$	30	37	10,780	26,299	TGF-β 0
2	$K_{t}=2$	9	14	235	5690	TGF-β 1
3	$K_{t}=3$	13	33	968	25,073	TGF-β 2
4	$K_{t}=4$	20	45	4726	29,508	TGF-β 3
5	$K_{u}=1$	46	35	28,737	25,928	ADAMTS16
6	$K_{u}=2$	17	34	3478	25,137	FGF21
7	$K_{u}=3$	52	39	40,048	28,152	TNFSF18
8	$K_{u}=4$	16	26	2467	19,160	ACAN
9	$K_{u}=5$	14	31	1489	24,511	RPH3A
10	$K_{u}=6$	42	46	26,600	29,559	ADAMTS8
11	$K_{u}=7$	51	47	34,769	39,960	MEGF6
12	$K_{u}=8$	40	38	26,295	27,326	SV2B
13	$K_{u}=9$	44	48	27,111	36,021	C1QTNF3
14	$K_{u}=10$	50	49	34,616	39,841	ANO4
15	$K_{u}=11$	32	24	12,696	16,566	IL11
16	$K_{u}=12$	43	30	26,624	23,640	CDH10
17	$K_{u}=13$	26	50	7263	30,243	HTR2B
18	$K_{u}=14$	19	16	4647	6551	LAMA1
19	$K_{u}=15$	28	36	8342	26,295	LAMA1
20	$K_{u}=16$	18	17	4021	8252	RAPGEF4
21	$K_{u}=17$	48	51	29,945	36,964	DNER
22	$K_{u}=18$	36	18	22,159	8569	GALNT3
23	$K_{u}=19$	47	23	29,145	15,531	ACSBG1
24	$K_{u}=20$	37	20	24,786	8735	OLFM2
25	$K_{d}=1$	35	40	19,039	28,262	CLEC3B
26	$K_{d}=2$	41	41	26,477	28,290	SCARA5
27	$K_{d}=3$	39	22	26,109	11,185	SLC10A6
28	$K_{d}=4$	24	44	6360	29,204	CXCL5
29	$K_{d}=5$	33	19	14,952	8729	MYOC
30	$K_{d}=6$	22	28	5961	22,288	IFITM1
31	$K_{d}=7$	21	13	5599	4483	ANGPTL4
32	$K_{d}=8$	38	25	25,538	17,434	SELENBP1
33	$K_{d}=9$	34	52	18,938	33,179	FMO1
34	$K_{d}=10$	49	53	34,080	39,427	GPR88
35	$K_{d}=11$	23	27	6276	22,141	HMGCS2
36	$K_{d}=12$	53	43	37,060	28,328	LGI2
37	$K_{d}=13$	29	11	9162	2485	PTN
38	$K_{d}=14$	11	15	513	5974	ADORA2A
39	$K_{d}=15$	27	29	7789	22,652	GFRA1
40	$K_{d}=16$	25	21	6718	8844	IL1R2
41	$K_{d}=17$	54	42	35,446	28,306	IL1R2
42	$K_{d}=18$	31	12	12,148	3444	PEG10
43	$K_{d}=19$	45	54	27,829	36,195	FMO2
44	$K_{d}=20$	15	32	1973	24,994	COX4I2
45	$K_{x}=1$	1	4	3	13	β-catenin
46	$K_{x}=2$	2	1	4	6	p53
47	$K_{x}=3$	3	2	11	10	ESR1
48	$K_{x}=4$	4	5	13	25	STAT3
49	$K_{x}=5$	5	3	22	11	RelA
50	$K_{x}=6$	6	6	38	82	PPAR-γ
51	$K_{x}=7$	7	8	111	767	IKK-β
52	$K_{x}=8$	8	7	179	198	SNAIL1
53	$K_{x}=9$	10	9	237	1520	MMP-14
54	$K_{x}=10$	12	10	578	2123	Flotillin-1

**Table 2:** List of 40 top protein pairs (a,b) with strongest sensitivity matrix element $D_{ab}$, with *a* belonging to the subgroups of up- or down-proteins and *b* belonging to the subgroups of TGF-β and X-proteins. The first column gives the ranking index $K_{s}$ of $D_{ab}$ matrix elements ordered by a decreasing value, the second to fourth columns provide the $K_{g},K_{u,d}$ indexes and the name of the protein (a), the fifth to seventh columns provide the $K_{g},K_{t,x}$ indexes and the name of the protein (b), and the eighth column shows the value of $D_{ab}$. See also Figure 5, which shows a color density plot for all matrix elements $D_{ab}$, and Table 1 for the list of considered proteins. An ordered list of all 560 values of sensitivity influence values $D_{ab}$ of TGF-β or X-proteins (for “*b*”) on up-/down proteins (for “*a*”) is available at [27].

$K_{s}$	$K_{g}\left(a\right)$	$K_{u,d}\left(a\right)$	Protein(a)	$K_{g}\left(b\right)$	$K_{t,x}\left(b\right)$	Protein(b)	$D_{\mathrm{ab}}$
1	25	$K_{d}=1$	CLEC3B	53	$K_{x}=9$	MMP-14	0.263109
2	22	$K_{u}=18$	GALNT3	46	$K_{x}=2$	p53	0.259298
3	13	$K_{u}=9$	C1QTNF3	50	$K_{x}=6$	PPAR-γ	0.225877
4	26	$K_{d}=2$	SCARA5	52	$K_{x}=8$	SNAIL1	0.219938
5	27	$K_{d}=3$	SLC10A6	50	$K_{x}=6$	PPAR-γ	0.214345
6	29	$K_{d}=5$	MYOC	54	$K_{x}=10$	Flotillin-1	0.200157
7	19	$K_{u}=15$	LAMA1	46	$K_{x}=2$	p53	0.199892
8	43	$K_{d}=19$	FMO2	47	$K_{x}=3$	ESR1	0.196550
9	29	$K_{d}=5$	MYOC	45	$K_{x}=1$	β-catenin	0.196394
10	39	$K_{d}=15$	GFRA1	45	$K_{x}=1$	β-catenin	0.184019
11	6	$K_{u}=2$	FGF21	46	$K_{x}=2$	p53	0.182339
12	20	$K_{u}=16$	RAPGEF4	45	$K_{x}=1$	β-catenin	0.182303
13	28	$K_{d}=4$	CXCL5	46	$K_{x}=2$	p53	0.181444
14	10	$K_{u}=6$	ADAMTS8	45	$K_{x}=1$	β-catenin	0.177848
15	42	$K_{d}=18$	PEG10	45	$K_{x}=1$	β-catenin	0.177726
16	35	$K_{d}=11$	HMGCS2	45	$K_{x}=1$	β-catenin	0.177443
17	15	$K_{u}=11$	IL11	45	$K_{x}=1$	β-catenin	0.177227
18	35	$K_{d}=11$	HMGCS2	46	$K_{x}=2$	p53	0.176906
19	21	$K_{u}=17$	DNER	45	$K_{x}=1$	β-catenin	0.176820
20	11	$K_{u}=7$	MEGF6	45	$K_{x}=1$	β-catenin	0.176612
21	36	$K_{d}=12$	LGI2	45	$K_{x}=1$	β-catenin	0.176606
22	7	$K_{u}=3$	TNFSF18	45	$K_{x}=1$	β-catenin	0.176603
23	41	$K_{d}=17$	IL1R2	45	$K_{x}=1$	β-catenin	0.176598
24	14	$K_{u}=10$	ANO4	45	$K_{x}=1$	β-catenin	0.176556
25	34	$K_{d}=10$	GPR88	45	$K_{x}=1$	β-catenin	0.176432
26	23	$K_{u}=19$	ACSBG1	45	$K_{x}=1$	β-catenin	0.176323
27	5	$K_{u}=1$	ADAMTS16	45	$K_{x}=1$	β-catenin	0.176315
28	12	$K_{u}=8$	SV2B	45	$K_{x}=1$	β-catenin	0.176264
29	17	$K_{u}=13$	HTR2B	45	$K_{x}=1$	β-catenin	0.176197
30	16	$K_{u}=12$	CDH10	45	$K_{x}=1$	β-catenin	0.176192
31	24	$K_{u}=20$	OLFM2	45	$K_{x}=1$	β-catenin	0.176038
32	32	$K_{d}=8$	SELENBP1	45	$K_{x}=1$	β-catenin	0.175939
33	33	$K_{d}=9$	FMO1	45	$K_{x}=1$	β-catenin	0.175776
34	33	$K_{d}=9$	FMO1	46	$K_{x}=2$	p53	0.175367
35	30	$K_{d}=6$	IFITM1	45	$K_{x}=1$	β-catenin	0.175056
36	44	$K_{d}=20$	COX4I2	45	$K_{x}=1$	β-catenin	0.174371
37	23	$K_{u}=19$	ACSBG1	46	$K_{x}=2$	p53	0.174167
38	34	$K_{d}=10$	GPR88	46	$K_{x}=2$	p53	0.173893
39	5	$K_{u}=1$	ADAMTS16	46	$K_{x}=2$	p53	0.173822
40	14	$K_{u}=10$	ANO4	46	$K_{x}=2$	p53	0.173770

**Table 3:** Values of the sum $D_{s}^{\left(u/d\right)}\left(b\right)=\sum_{a=5}^{44}D_{ab}$ (i.e., the *a*-sum is over up- and down-proteins) for *b* belonging to the TGF-β or the X-proteins subgroups. The list is ordered with respect to decreasing $D_{s}^{\left(u/d\right)}\left(b\right)$ values with the first column giving the corresponding ranking index; the second and third columns giving the $K_{g},K_{t,x}$ indexes; the fourth and fifth columns containing the local PageRank index *K* and the name of the protein *b*; and the sixth and seventh columns giving the values of $D_{s}^{\left(u/d\right)}\left(b\right)$ and the local PageRank probability $P_{r}\left(b\right)$. Both *K* and $P_{r}\left(b\right)$ correspond to the group of 54 fibrosis proteins of Table 1.

Rank	$K_{g}\left(b\right)$	$K_{t,x}\left(b\right)$	*K*	Protein (b)	$D_{s}^{\left(u/d\right)}\left(b\right)$	$P_{r}\left(b\right)$
1	45	$K_{x}=1$	1	β-catenin	6.809993	0.175768
2	46	$K_{x}=2$	2	p53	6.789229	0.171249
3	47	$K_{x}=3$	3	ESR1	4.513399	0.113285
4	48	$K_{x}=4$	4	STAT3	4.109638	0.104088
5	49	$K_{x}=5$	5	RelA	3.343309	0.085443
6	50	$K_{x}=6$	6	PPAR-γ	3.086237	0.070668
7	51	$K_{x}=7$	7	IKK-β	1.696330	0.043249
8	52	$K_{x}=8$	8	SNAIL1	1.477019	0.034269
9	53	$K_{x}=9$	10	MMP-14	1.368302	0.029121
10	2	$K_{t}=2$	9	TGF-β 1	1.081828	0.029166
11	54	$K_{x}=10$	12	Flotillin-1	0.787569	0.016863
12	3	$K_{t}=3$	13	TGF-β 2	0.333981	0.012451
13	4	$K_{t}=4$	20	TGF-β 3	0.159633	0.004157
14	1	$K_{t}=1$	30	TGF-β 0	0.081261	0.002090

## Appendix A

### Appendix A.1. Additional Figures for REGOMAX Results

Here we present additional Appendix Figure A1, Figure A2, Figure A3, Figure A4 snd Figure A5 for the main part of this article.

Appendix AFigure A1 provides complementary information to Figure 1.

Appendix AFigure A2 provides the network diagrams similar as in Figure 4 but with a different choice of 5 top nodes based on the criterion of top positions in Table 2.

Appendix AFigure A3 shows the sensitivity matrix $D_{ab}^{\left(166\right)}$ for the intermediary group of 166 proteins which was used to determine the additional 10 X-proteins as explained in Section 2.6.

Appendix AFigure A4 provides an additional analysis of the overall influence of the TGF-β proteins on the up- and down-proteins which is discussed in Section 3.5.

Appendix Figure A5 provides the graphical and fit verification of the linear behavior between the two quantities $D_{s}^{\left(u/d\right)}\left(b\right)$ and $P_{r}\left(b\right)$ appearing the last two columns of Table 3.

### Appendix A.2. Simple Estimate for the Sensitivity Matrix

In the second part of this appendix we remind some details (see [34]) about the numerical computation of the sensitivity (8) and provide an analytic approximation based on a simplified model. Let (a,b) be an arbitrary index pair and $G_{\varepsilon }$ be the perturbed Google matrix obtained from a general unperturbed Google matrix $G_{0}$ by multiplying its element $G_{0}\left(a,b\right)$ at position (a,b) by (1+ε) and then sum-renormalizing the column *b* to unity. The elements in the other columns are not modified. In a more explicit formula we have:

(A1) $$\forall_{c,d}\;G_{\varepsilon }\left(c,d\right)=\frac{\left(1+\varepsilon \;\delta_{ca}\delta_{db}\right)\;G_{0}\left(c,d\right)}{1+\varepsilon \;\delta_{db}\;G_{0}\left(a,b\right)}$$

where $\delta_{ca}=1$ (or 0) if c=a (or c≠a). Note that the denominator is either 1 if d≠b or the modified column sum $1+\varepsilon \;G_{0}\left(a,b\right)$ of column *b* if d=b. Expanding (A1) up to first order in ε we obtain $G_{\varepsilon }=G_{0}+\varepsilon \Delta G+\ldots$ with ΔG having the elements:

(A2) $$\forall_{c,d}\;\Delta G\left(c,d\right)=\delta_{ca}\delta_{db}\;G_{0}\left(c,d\right)-\delta_{db}\;G_{0}\left(a,b\right)\;G_{0}\left(c,d\right)\;.$$

Let $P_{\varepsilon }$ be the sum-normalized PageRank vector of $G_{\varepsilon }$ determined by the conditions $G_{\varepsilon }\;P_{\varepsilon }=P_{\varepsilon }$ and the normalization $E^{T}\;P_{\varepsilon }=1$ where $E^{T}=\left(1,\ldots ,1\right)$ is a (row) vector with unit entries. Note that the column sum condition of $G_{\varepsilon }$ can be written as $E^{T}\;G_{\varepsilon }=E^{T}$ and of course for ε=0 we also have $G_{0}\;P_{0}=P_{0}$, $E^{T}\;P_{0}=1$ and $E^{T}\;G_{0}=E^{T}$. Furthermore we write the perturbed PageRank vector in the form $P_{\varepsilon }=P_{0}+\varepsilon \Delta P+\ldots$ where the ΔP must satisfy the condition $E^{T}\;\Delta P=0$. Then the sensitivity (8) is directly related to ΔP by:

(A3) $$D_{\left(b\to a\right)}\left(j\right)=\frac{\Delta P(j)}{P_{0}\left(j\right)}\;.$$

Expanding the PageRank equation $G_{\varepsilon }\;P_{\varepsilon }=\left(G_{0}+\varepsilon \Delta G+\ldots \right)\left(P_{0}+\varepsilon \Delta P+\ldots \right)=P_{\varepsilon }=P_{0}+\varepsilon \Delta P+\ldots$ to order one we first obtain the unperturbed PageRank equation $G_{0}\;P_{0}=P_{0}$ and a further inhomogeneous equation:

(A4) $$\Delta P=G_{0}\;\;\Delta P+\Delta G\;\;P_{0}$$

which can be efficiently numerically solved by iteration (choosing initially ΔP=0 on the right hand side) once $P_{0}$ has been computed (see [34] for details on this point). This provides a numerical precise scheme to compute the sensitivity in the limit ε→0 without the need to take finite ε-differences.

Now, we consider a particular very simple model where $G_{0}$ has identical columns being the PageRank $P_{0}$, i.e., $G_{0}=P_{0}\;E^{T}$ or more explicitely $G_{0}\left(c,d\right)=P_{0}\left(c\right)$ for all values of c,d. Then we obtain from (A2)

(A5) $$\forall_{c,d}\;\Delta G\left(c,d\right)=\delta_{ca}\delta_{db}\;P_{0}\left(c\right)-\delta_{db}\;P_{0}\left(a\right)\;P_{0}\left(c\right)$$

and from (A4)

(A6) $$\Delta P=\left(P_{0}\;E^{T}\right)\;\Delta P+\Delta G\;P_{0}=\Delta G\;P_{0}$$

since $E^{T}\;\Delta P=0$. Inserting (A5) in (A6) we obtain (replacing c=j and performing the *d*-sum for the matrix vector product)

(A7) $$\forall_{j}\;\Delta P\left(j\right)=\left[\delta_{ja}-P_{0}\left(a\right)\right]\;P_{0}\left(j\right)\;P_{0}\left(b\right)$$

and from (A3)

(A8) $$D_{\left(b\to a\right)}\left(j\right)=\left[\delta_{ja}-P_{0}\left(a\right)\right]\;P_{0}\left(b\right)\;.$$

Choosing j=a this gives the sensitivity matrix

(A9) $$D_{ab}=D_{\left(b\to a\right)}\left(a\right)=\left[1-P_{0}\left(a\right)\right]\;P_{0}\left(b\right)\approx P_{0}\left(b\right)$$

where the last approximation holds if typically $P_{0}\left(a\right)\ll 1$.

This result is of course only valid for the simplified model of identical columns (being the PageRank vector) in $G_{0}$. However, when $G_{0}$ represents a typical reduced Google matrix, with $N_{r}\ll N$, the component $G_{\mathrm{pr}}$ which has the strongest numerical weight (typically ∼95%) is of the form $G_{\mathrm{pr}}={\tilde{P}}_{0}\;{\tilde{E}}^{T}$ where ${\tilde{P}}_{0}\approx P_{0}$ and ${\tilde{E}}^{T}\approx E^{T}$ except for a few number of components *j* where strong deviations between ${\tilde{P}}_{0}\left(j\right)$ and $P_{0}\left(j\right)$ (and similarly between $\tilde{E}\left(j\right)$ and E(j)) are possible.

Our examples of $D_{ab}$ visible in Figure 5 and of $D_{ab}^{\left(166\right)}$ of Appendix A Figure A3 confirm the typical behavior $D_{ab}∼P_{0}\left(b\right)$ for a “uniform background” but there are some exceptional peak values which arise from the deviations from $G_{\mathrm{pr}}$ to the simplified model and also from the contributions of $G_{\mathrm{rr}}$ and $G_{\mathrm{qr}}$. This also explains our numerical finding that all matrix elements of $D_{ab}$ are positive. Actually, according to (A8) we expect that $D_{\left(b\to a\right)}\left(j\right)$ is typically positive if j=a and negative if j≠a.

Furthermore, when taking the partial *a*-sum over up- and down-nodes of $D_{ab}$ the effect of exceptional peaks is strongly reduced thus explaining the linear behavior $D_{s}^{\left(u/d\right)}\left(b\right)\approx \eta P_{r}\left(b\right)$ visible in Table 3 and Figure A5.

## References

[1] Murtha L.A., Schuliga M.J., Mabotuwana N.S., Hardy S.A., Waters D.W., Burgess J.K., Knight D.A., Boyle A.J. (2017). The processes and mechanisms of cardiac and pulmonary fibrosis. Front. Physiol..

[2] Liu T., Song D., Dong J., Zhu P., Liu J., Liu W., Ma X., Zhao L., Ling S. (2017). Current understanding of the pathophysiology of myocardial fibrosis and its quantitative assessment in heart failure. Front. Physiol..

[3] Meng X., Nikolic-Paterson D., Lan H. (2016). TGF-*β*: The master regulator of fibrosis. Nat. Rev. Nephrol..

[4] Wynn T.A. (2017). Cellular and molecular mechanisms of fibrosis. J. Pathol..

[5] Pintus S.S., Sharipov R.N., Kel A., Timotin A., Keita S., Martinelli I., Boal F., Tronchere H., Kolpakov F., Kunduzova O. (2021). Drug repositioning for cardiac fibrosis through molecular signature of aberrant fibroblast activation. INSERM Prepr..

[6] Karimizadeh E., Sharifi-Zarchi A., Nikaein H., Salehi S., Salamatian B., Elmi N., Gharibdoost F., Mahmoudi M. (2019). Analysis of gene expression profiles and protein-protein interaction networks in multiple tissues of systemic sclerosis. BMC Med. Genom..

[7] Pchejetski D., Foussal C., Alfarano C., Lairez O., Calise D., Guilbeau-Frugier C., Schaak S., Seguelas M.-H., Wanecq E., Valet P. (2012). Apelin prevents cardiac fibroblast activation and collagen production through inhibition of sphingosine kinase 1. Eur. Heart J..

[8] MetaCore. https://clarivate.com/cortellis/solutions/early-research-intelligence-solutions/.

[9] Ekins S., Bugrim A., Brovold L., Kirillov E., Nikolsky Y., Rakhmatulin E., Sorokina S., Ryabov A., Serebryiskaya T., Melnikov A. (2006). Algorithms for network analysis in systems-ADME/Tox using the MetaCore and MetaDrug platforms. Xenobiotica.

[10] Bessarabova M., Ishkin A., JeBailey L., Nikolskaya T., Nikolsky Y. (2012). Knowledge-based analysis of proteomics data. BMC Bioinform..

[11] Kotelnokova E., Frahm K.M., Lages J., Shepelyansky D.L. (2021). Statistical properties of the MetaCore network of protein-protein interactions. bioRxiv.

[12] Baek M., DiMaio F., Anishchenko I., Dauparas J., Ovchinnikov S., Lee G.R., Wang J., Cong Q., Kinch L.N., Schaeffer R.D. (2021). Accurate prediction of protein structures and interactions using a three-track neural network. Science.

[13] TRANSPATH. https://genexplain.com/transpath/.

[14] REACTOME. https://reactome.org/.

[15] Brin S., Page L. (1998). The anatomy of a large-scale hypertextual Web search engine. Comput. Netw. ISDN Syst..

[16] Langville A.M., Meyer C.D. (2006). Google’s PageRank and Beyond: The Science of Search Engine Rankings.

[17] Ermann L., Frahm K.M., Shepelyansky D.L. (2015). Google matrix analysis of directed networks. Rev. Mod. Phys..

[18] Markov A.A. (1971). Rasprostranenie Zakona Bol’shih Chisel na Velichiny, Zavisyaschie Drug ot Druga, Izvestiya Fiziko-Matematicheskogo Obschestva pri Kazanskom Universitete, 2-ya Seriya (in Russian) 15 (1906) 135; English Translation: Extension of the Limit Theorems of Probability Theory to a Sum of Variables Connected in a Chain, Reprinted in Appendix B of: Howard R., Dynamic Probabilistic Systems 1: Markov Chains.

[19] Frahm K.M., Shepelyansky D.L. (2016). Reduced Google matrix. arXiv.

[20] Frahm K.M., Jaffres-Runser K., Shepelyansky D.L. (2015). Wikipedia mining of hidden links between political leaders. Eur. Phys. J. B.

[21] Lages J., Shepelyansky D.L., Zinovyev A. (2018). Inferring hidden causal relations between pathway members using reduced Google matrix of directed biological networks. PLoS ONE.

[22] Frahm K.M., Shepelyansky D.L. (2020). Google matrix analysis of bi-functional SIGNOR network of protein-protein interactions. Phys. A.

[23] Rollin G., Lages J., Shepelyansky D.L. (2019). World influence of infectious diseases from Wikipedia network analysis. IEEE Access.

[24] Rollin G., Lages J., Shepelyansky D.L. (2019). Wikipedia network analysis of cancer interactions and world influence. PLoS ONE.

[25] Coquide C., Ermann L., Lages J., Shepelyansky D.L. (2019). Influence of petroleum and gas trade on EU economies from the reduced Google matrix analysis of UN COMTRADE data. Eur. Phys. J. B.

[26] Coquide C., Lages J., Shepelyansky D.L. (2020). Interdependence of sectors of economic activities for world countries from the reduced Google matrix analysis of WTO data. Entropy.

[27] http://www.quantware.ups-tlse.fr/QWLIB/fibrosisPPInetwork/.

[28] Chepelianskii A.D. (2010). Towards physical laws for software architecture. arXiv.

[29] Zhirov A.O., Zhirov O.V., Shepelyansky D.L. (2010). Two-dimensional ranking of Wikipedia articles. Eur. Phys. J. B.

[30] Fushen Z. (2005). The Schur Complement and Its Applications.

[31] Beenakker C.W.J. (1997). Random-Matrix Theory of Quantum Transport. Rev. Mod. Phys..

[32] Gaspard P. (2014). Quantum chaotic scattering. Scholarpedia.

[33] Meyer C.D. (1989). Stochastic complementation, uncoupling Markov chains, and the theory of nearly reducible systems. SIAM Rev..

[34] Frahm K.M., Shepelyansky D.L. (2019). Linear response theory for Google matrix. arXiv.

